# The major diagnostic VSG LiTat 1.3 of the human parasite *Trypanosoma brucei gambiense* is a trimer in solution

**DOI:** 10.1371/journal.ppat.1014530

**Published:** 2026-08-10

**Authors:** Niki Danel, Rob Geens, Israel Mares-Mejía, Wim Versées, Jan Van Den Abbeele, Yann G. J. Sterckx

**Affiliations:** 1 Trypanosoma Unit, Department of Biomedical Sciences, Institute of Tropical Medicine, Nationalestraat, Antwerp, Belgium; 2 VIB-VUB Center for Structural Biology, VIB, Pleinlaan 2, Brussels, Belgium; 3 Structural Biology Brussels, Vrije Universiteit Brussel, Pleinlaan 2, Brussels, Belgium; 4 Laboratory of Medical Biochemistry and the Infla-Med Centre of Excellence, University of Antwerp, Universiteitsplein1, Wilrijk, Belgium; University of Geneva Faculty of Medicine: Universite de Geneve Faculte de Medecine, SWITZERLAND

## Abstract

Human African trypanosomiasis (HAT) remains a significant health burden in sub-Saharan Africa, with serological diagnosis relying heavily on parasite variant surface glycoproteins (VSGs). Experimentally validated structural studies of VSGs from *Trypanosoma brucei gambiense* (the main HAT causative agent) are currently lacking despite their diagnostic importance. In this study, we present a thorough characterization of LiTat 1.3, a key VSG in the diagnosis of *T. b. gambiense* infections, through an integrative structural biology approach encompassing AlphaFold-based structure prediction, analytical gel filtration (AGF), size exclusion chromatography with multi-angle light scattering (SEC-MALS), and small-angle X-ray scattering (SAXS). While sequence-based analyses had previously proposed LiTat 1.3 as a homotrimeric B2 class VSG, experimental evidence was yet to be presented. Here, we demonstrate that LiTat 1.3 displays a stable, concentration-independent homotrimeric architecture in solution, which is distinct from other trimeric *T. brucei brucei* VSGs described to date. Furthermore, the SAXS data demonstrate that the C-terminal domains of trimeric VSGs exhibit the same degree of flexibility as observed in dimeric VSGs. Hence, the biophysical characterization of LiTat 1.3 VSG adds to the growing body of knowledge that certain VSG classes occur as homotrimers instead of homodimers. Together, these results not only expand our knowledge of VSG structure and flexibility but also provide the first detailed structural insight into a major diagnostic VSG of *T. b. gambiense*, which holds potential for understanding antibody recognition and guiding the development of improved serological tests.

## Author summary

Human African trypanosomiasis (HAT) is mainly caused by *Trypanosoma brucei gambiense*, a parasite transmitted by tsetse flies. To survive in the human host, these parasites cover themselves with a coat consisting of millions of identical copies of surface proteins called variant surface glycoproteins (VSGs). This VSG coat is regularly switched by the parasite to escape the immune system. Some of these VSGs, including one known as LiTat 1.3, are used in diagnostic tests to detect potentially infected patients. In our study, we discovered that LiTat 1.3 assembles into groups of three identical molecules (homotrimers). Using structural and biophysical techniques, we showed that this trimeric form is stable in solution and retains the dynamic behavior observed in dimeric VSGs. Understanding how such structural variations arise and how they influence immune recognition may help explain why certain VSGs, like LiTat 1.3, are particularly effective in diagnosis and could ultimately guide the development of improved tools to monitor and control sleeping sickness.

## Introduction

Human and animal African trypanosomiasis (HAT and AAT, respectively) are neglected tropical diseases caused by protozoan parasites of the *Trypanosoma* genus. The *T. brucei* species harbors three subspecies: *T. b. gambiense* and *T. b. rhodesiense* (both human-infective and responsible for HAT, alias sleeping sickness) and *T. b. brucei* (non-infective to humans and cause of AAT, alias nagana). Since transmission solely relies on blood-feeding tsetse flies (*Glossina* spp.), the geographical range of these parasites is correlated with the spread of the insect vector, which is typically confined to foci in sub-Saharan Africa. While *T. b. rhodesiense* causes acute infections, *T. b. gambiense* (gHAT) cases are chronic and responsible for 94% of reported HAT cases in 2024. Infection by either subspecies leads to fatal neurological symptoms if left untreated [1,2].

African trypanosomes are extracellular parasites that can survive in their vertebrate hosts for months or even years, despite being continuously exposed to the immune system. Once metacyclic trypomastigotes are inoculated into the vertebrate host through the bite of an infected tsetse fly, the parasites transform into bloodstream forms (BSFs) giving rise to a systemic infection. The BSFs thrive within the host blood circulation but they also colonize the lymphatics, skin, brain, testes, adipose tissue, and lungs [3–7]. Being extracellular parasites, BSFs rely on sophisticated mechanisms to evade host immune responses. Among these, the antigenic variation is the most notorious in which trypanosomes employ a controlled mechanism of switching their major surface antigens called variant surface glycoproteins (VSGs) [8–12]. Although trypanosomes have a repertoire of >1000 VSG-encoding genes, individual *T. brucei* parasites only express a single gene at a time, thereby resulting in the display of identical VSGs at their surface. On the latter, the expressed VSGs form a dense monolayer of around 10^7^ identical molecules that act as a protective barrier to shield underlying invariant proteins from immune recognition. Each VSG coat is called a variant antigen type (VAT), and trypanosomes expressing the same VSG belong to the same VAT. VSGs are highly immunogenic and readily elicit an effective humoral response, in which antibodies matured against the exposed VAT lead to parasite clearance. Antigenic variation entails that trypanosomes switch their VSG coat by successively cycling their *VSG* gene repertoire, either by activating a different intact *VSG* gene or by generating novel mosaic VSGs. This leads to the emergence of parasite subpopulations that escape recognition by existing antibodies, giving rise to new parasitemic waves [12,13].

The structure-function relationship of VSGs has received much attention given their importance in trypanosome immunobiology. Despite a low amino acid sequence identity, all VSGs possess a conserved structural architecture: i) an N-terminal domain (NTD; 300–400 residues) consisting of a membrane-distal top lobe separated from a membrane-proximal bottom lobe by a 3-helix stalk, and ii) a C-terminal domain (CTD; 80–120 residues) harboring a glycosylphosphatidylinositol (GPI) anchor, which attaches the VSG to the parasite surface [14]. VSG NTDs are further categorized into two classes (A and B) based on the arrangement of the bottom lobe in their primary sequences. Class A includes all VSGs studied to date that form homodimers, with their bottom lobe residues positioned at the C-terminal end of the NTD. In contrast, class B comprises VSGs that form concentration-dependent homotrimers, with their bottom lobe residues located at the N-terminal end. Furthermore, class B VSGs frequently feature *O*-linked carbohydrate modifications, a trait that has not yet been observed in class A VSGs [14–16]. Hence, three features of VSG structure underpin antigenic variation: i) the NTDs display poor sequence conservation (~10–30% sequence identity) and adopt distinct structural topologies corresponding to the major VSG classes, ii) VSGs may undergo post-translational modifications (e.g., *O*-linked glycosylation) with potent immunomodulatory effects [14–16], challenging the belief that antigenic variation is solely driven by amino acid sequence divergence, and iii) more recently, VSGs have been shown to adopt distinct oligomeric states (thereby challenging the longstanding view that VSGs solely occur as homodimers). Studies on the solution structure of homodimeric VSGs have revealed that, while the NTDs behave as rigid entities, the CTDs are hallmarked by a high degree of flexibility, thereby rendering the entire VSG coat highly dynamic [17,18]. While the same behavior could be expected for homotrimeric VSGs, such evidence is yet to be presented.

As the clinical signs of gHAT are not specific enough for an unambiguous diagnosis, sensitive and specific diagnosis plays a key role in identifying a trypanosome positive case. Since the 1970s, serological tests relying on the detection of VAT-specific antibodies have formed the cornerstone of gHAT diagnosis and are critical for the successful reduction in disease incidence during the last decades. These serological gHAT diagnostic tests all rely on antibody reactivity towards two *T. b. gambiense* VSGs: the Lille Trypanozoon antigen types 1.3 and 1.5 (LiTat 1.3 and LiTat 1.5, respectively) [19–21]. Given their key-role for gHAT serological screening/diagnosis, an in-depth knowledge of the solution state structure of these *T. b. gambiense* VSGs represents an added value. While LiTat 1.5 was recently characterized as an archetypical dimeric class A1 VSG [18], the structural details of LiTat 1.3 remain poorly understood. While sequence-based analyses had previously proposed LiTat 1.3 as a homotrimeric B2 class VSG [22], this is yet to be experimentally validated. Here, we present the application of an integrative structural biology approach to investigate the solution state of *T. b. gambiense* LiTat 1.3 VSG. By combining AlphaFold structural modeling, analytical gel filtration (AGF), size exclusion chromatography coupled to multi-angle light scattering (SEC-MALS) and small-angle X-ray scattering (SAXS), our findings provide compelling evidence that LiTat 1.3 exists as a homotrimer in solution. Importantly, our data show that LiTat 1.3 forms a stable homotrimer in solution even at low protein concentrations, in contrast to previously studied B-type VSGs, where trimerization has been observed to be concentration-dependent. Furthermore, the SAXS-based modeling shows that the LiTat 1.3 solution behavior is best described by a conformational ensemble due to a highly flexible CTD, thereby indicating that the CTDs found in trimeric VSGs exhibit the same degree of structural plasticity as those present in VSG dimers. Our results therefore suggest that trimeric VSGs also contribute to the parasite’s highly dynamic surface antigen coat.

## Materials and methods

### Ethics statement

Experimental protocols for the *in vivo* cultivation of *T. b. gambiense* were reviewed and approved by the Ethical Committee of the Institute of Tropical Medicine, Antwerp, Belgium (approval codes TTP-2021–1 and TTP-2021–2).

### Protein production and purification

The purified native VSG LiTat 1.3 was provided by the Applied Technology and Production unit at ITM and was part of a large VSG batch that was used for the production of the gHAT-RDT diagnostic test (HAT Sero K-SeT) by Coris BioConcept (Gembloux). To initiate the VSG production, *T. b. gambiense* bloodstream forms expressing LiTat 1.3 were cultivated in rats after intraperitoneal injection of ~5 x 10^7^ trypanosomes/mL, administered at 1 mL per 100 g body weight. Approximately 72 hours after initiating the rat infection, when parasites are in their first expanding growth phase, blood was collected and passed over a self-packed diethylaminoethyl (DEAE)-52 column (8 L bed volume) to retain the trypanosomes. Following sample loading, the column was washed with phosphate-saline-glucose (PSG; 83 mM D-(+)-Glucose, 38 mM Na_2_HPO_4_, 29 mM NaCl, 2 mM NaH_2_PO_4_, pH 8.0) until the eluate was clear. Elution was carried out at a fixed flow rate (maintained at approximately one drop per second) using a peristaltic pump and the collected trypanosome-containing fraction was kept on ice. Subsequently, the trypanosome suspension was concentrated and subjected to two washing steps in PSG via sequential centrifugation (1,000 x g and 4°C). The concentrated parasite suspension was aliquoted and centrifuged again. Following the removal of the supernatant, the resulting 1 mL pellets were stored at -80°C.

For VSG extraction and purification, the trypanosome pellets were resuspended in five volumes of ice-cold Concanavalin A (ConA) binding buffer (500 mM NaCl, 1 mM MgCl₂ 1 µM CaCl₂, 10% (v/v) phosphate buffer (100 mM Na₂HPO₄ titrated with 100 mM NaH₂PO₄ to pH 8.0) and transferred to 10 mL centrifuge tubes on ice. The suspension was vortexed to ensure homogeneity, incubated on ice for 15 min, and centrifuged (42,000 x g for 2 h at 4°C). The supernatant was transferred to a new tube, and the pellet was resuspended in 3 mL of ice-cold ConA binding buffer, vortexed and centrifuged again. The resulting supernatants were combined, filtered (0.22 µm filter), and stored at 4°C. The next day, the VSG-containing supernatant was loaded onto a 1 mL HiTRAP ConA 4B column (Cytiva) at a flow rate of 0.5 mL/min, after which the column was washed with 10 column volumes (CVs) of ConA binding buffer, and soluble VSG was eluted using ConA elution buffer (ConA binding buffer supplemented with 10% methyl α-D-mannopyranoside). ConA affinity chromatography is a routinely used strategy for isolating soluble native VSG from trypanosome surface coats and does not change the native quaternary structure of the protein [23]. Then, the VSG eluate was concentrated to approximately 10 mg/mL and subsequently buffer switched to either HEPES-buffered saline (HBS) or phosphate-buffered saline (PBS), using a 30-kDa cutoff centrifugal filter (Centricon) and stored at -80°C.

### Analytical gel filtration

Analytical gel filtration (AGF) was performed using a Superdex 200 Increase GL 10/300 column (Cytiva) coupled to an ÄKTA FPLC system. The column was pre-equilibrated with HBS (25 mM HEPES, 150 mM NaCl, pH 7.4). Samples of 100 µL were injected and eluted at a flow rate of 0.75 mL/min. The Bio-Rad gel filtration standard was run under the same conditions for molecular mass estimation. Relevant elution fractions were collected for SDS-PAGE analysis and stored at -80°C.

### SDS-PAGE

Purified VSG samples were mixed with 4x SDS sample buffer and heated at 100°C for 10 min to ensure complete denaturation. Samples were loaded on a 4–15% mini-PROTEAN TGX precast gel (Bio-Rad) and electrophoresis was performed in Tris-Glycine-SDS buffer (Bio-Rad) at 120 V. Protein bands were visualized using PageBlue protein staining solution (Thermo Scientific) with the Precision Plus Protein Dual Color standard (Bio-Rad) allowing molecular mass estimations.

### Small-angle X-ray scattering and ensemble modelling

Small-angle X-ray scattering (SAXS) was conducted at the BioSAXS beamline SWING (SOLEIL, Gif-sur-Yvette, France) [24]. SEC-SAXS data were acquired on a Shodex KW404-4F column, pre-equilibrated in 20 mM HEPES, 150 mM NaCl, 3% glycerol, pH 7.4. A sample of 50 µL (15.51 mg/mL) was injected and subsequently eluted at a flow rate of 0.2 mL/min. Scattering data were collected with an exposure time of 750 msec and a dead time of 10 msec, calibrated to absolute units using the scattering of pure water [25]. Data processing and analysis was performed using the ATSAS package and BioXTAS RAW [26–28].

Molecular models of LiTat 1.3 dimers and trimers were generated using AlphaFold-Multimer [29] based on the primary amino acid sequence (GenBank accession no. KJ499460.1) [30]. The signal peptide, which is cleaved from the mature VSG protein, was predicted using the SignalP 6.0 tool [31] and excluded from subsequent modeling. In addition, the C-terminal GPI-anchor signal sequence was predicted using NetGPI-1.1 and likewise excluded prior to modeling [32]. Theoretical scattering curves of the AlphaFold2 models and their respective fits to the experimental data were calculated using FoXS [33]. SAXS-based ensemble modelling was carried out using BilboMD [34,35]. All simulations were performed using the default BilboMD pipeline. Briefly, conformational sampling was performed using the standard CHARMM-based implementation. Following energy minimization, high-temperature molecular dynamics simulations at 1500 K were performed in vacuum, with only the atoms of the flexible domains allowed to move. The resulting conformers were validated using FoXS followed by minimal ensemble selection with MultiFoXS [34–36]. BilboMD Classic was used to generate conformational ensembles by sampling six R_g_ bins, with minimal and maximal R_g_ values set at 7% and 35% of the experimentally determined R_g_, respectively. For each R_g_ bin, 800 conformations were generated, yielding 4800 conformers per BilboMD Classic run. In total, three independent BilboMD Classic runs were performed, and the resulting pools were subsequently combined into a single super-pool (14400 conformers) from which a minimal ensemble was selected using BilboMD Multi and the experimental scattering curve [34]. The overall goodness-of-fit between the final models and the experimental data are reported through the calculation of a χ2 value, with N_k_ being the number of points, σ(q_j_) the standard deviations, and c a scaling factor.


$$\begin{array}{c} \chi^{\text{2}}=\frac{\text{1}}{N_{k}}\cdot \sum_{j=\text{1}}^{N_{k}}{\left[\frac{I_{exp}\left(q_{j}\right)-c\cdot I_{calc}\left(q_{j}\right)}{\sigma \left(q_{j}\right)}\right]}^{\text{2}} \end{array}$$

Furthermore, comparisons of theoretical and experimental scattering curves include the presentation of a residuals plot (Δ/σ vs. q, where Δ indicates the difference between experimental and calculated intensities), which enables a local inspection of the model fit to the data. The information on data collection, scattering-derived structural parameters, and modeling is summarized in S1 Table. All SAXS data have been deposited in the SASBDB [37] under accession code SASDXA9. The structural ensemble has been deposited to the Protein Ensemble Database (PED) [38] with the accession number PED07322. Specific ensemble metrics are also reported in S1 Table.

Intrinsic disorder was predicted from the LiTat 1.3 amino acid sequence using Metapredict [39], IUPred3 [40], AIUPred [41] and PrDOS [42]. Visualization and structural analysis of molecular models were conducted using UCSF ChimeraX [43].

### Size exclusion chromatography with multi-angle light scattering

Purified VSG LiTat 1.3 was analyzed by size exclusion chromatography using an Agilent Bio SEC-3 column coupled to an HPLC Alliance system (Waters) equipped with a 2998 PDA detector (Waters), a TREOS II MALS detector (Wyatt technology) and a RI-501 refractive index detector (Shodex). The samples were prepared at two concentrations (0.5 mg/mL and 5 mg/mL) in PBS. A volume of 50 µL of each was injected onto a pre-equilibrated column in the same buffer at a constant flow rate of 0.2 mL/min. The Astra 7.3.0 software (Wyatt Technology) was used to analyze the data, and a BSA sample (1 mg/mL) was used to normalize and align the signals of the different detectors, using a dn/dc value of 0.185 mL/g.

## Results

### *In silico* structure predictions of LiTat 1.3 VSG favor a homotrimeric arrangement

**Fig 1:**
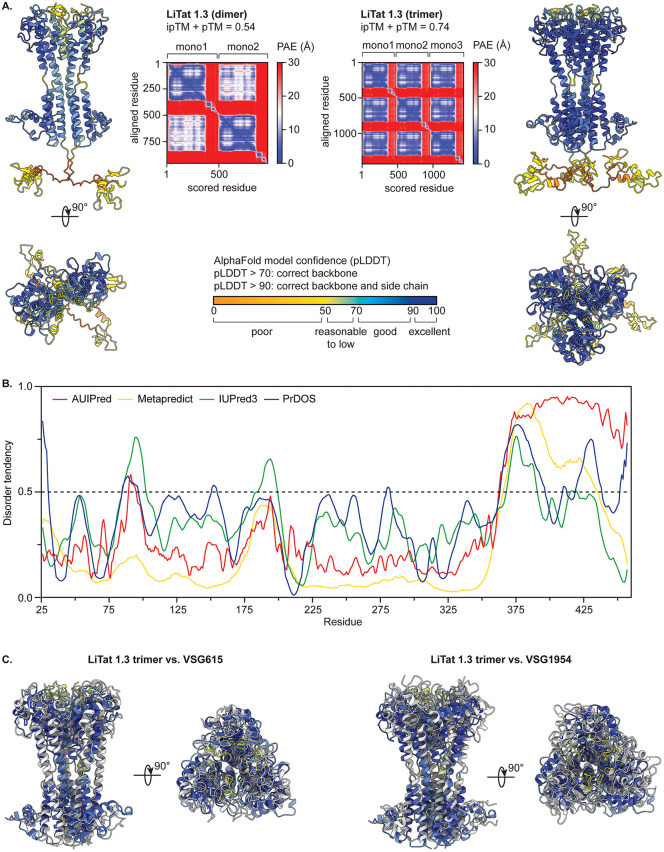
AlphaFold-based structure prediction of LiTat 1.3 and analysis of the obtained models. (A.) Cartoon representations of the LiTat 1.3 dimer (left) and trimer (right) models generated by AlphaFold. The models are colored according to the predicted local distance difference test (pLDDT) score, which reflects (local) model quality as indicated by the legend at the bottom. For both models, the predicted aligned error (PAE) and AlphaFold-Multimer model confidence (0.8*ipTM + 0.2*pTM) are shown. The PAE provides a distance error for every residue pair and is calculated for each residue x (scored residue) when the predicted and true structures are aligned on residue y (aligned residue). The pTM (score between 0 and 1) provides a measure of similarity between two protein structures (in this case, the predicted and unknown true structure) over all residues and thus reports on the accuracy of prediction within a single chain. The interface pTM (ipTM, score between 0 and 1) provides a measure of similarity between two protein structures (in this case, the predicted and unknown true structure) over only interfacing residues and thus reports on the accuracy of prediction for a complex. (B.) Disorder tendency of full-length LiTat 1.3 VSG (sequence devoid of signal peptide and GPI anchor) predicted by several algorithms. Residues with disorder tendencies above the threshold of 0.5 (dotted line) are considered disordered. (C.) Structural overlay of the NTDs of i) the AlphaFold LiTat 1.3 trimer model and the experimentally determined structure for VSG615 (left), and ii) the AlphaFold LiTat 1.3 trimer model and the experimentally determined structure for VSG1954 (right). In both cases, the AlphaFold LiTat 1.3 model is colored according to the pLDDT score as in panel A, while the experimentally determined structures are colored in light gray.

The LiTat 1.3 NTD was first classified using a Hidden Markov Model (HMM)-based Python pipeline [44], which automatically assigns the most probable subtype (A1–A3, B1–B2) based on the highest-scoring sequence alignment against HMM profiles derived from previously typed VSGs. Consistent with earlier work of So et al. 2022 [22], our HMM-based analysis also assigned LiTat 1.3 to the B2 subtype, which harbors trimeric VSGs [14]. Next, we employed AlphaFold-Multimer to predict structural models for both dimeric and trimeric configurations of LiTat 1.3. Consistent with the assignment of LiTat 1.3 as a B type VSG, a comparative analysis reveals that the trimeric structural model is favored, as it is supported by superior local and global validation metrics (Fig 1A). Especially the NTDs and their interaction interfaces are predicted with great confidence as advocated by the predicted aligned error (PAE) plots and AlphaFold-Multimer confidence scores. Interestingly, the CTDs are characterized by significantly lower pLDDT scores, which implies that these may be intrinsically disordered [45]. This was further assessed by performing sequence-based intrinsic disorder predictions using multiple algorithms (Fig 1B). All predictors identify the CTD of LiTat 1.3 as highly disordered, whereas the NTD is predominantly predicted to be structured. Finally, a structural superposition of the LiTat 1.3 trimer AlphaFold model with crystal structures of other Class B2 VSGs [14,16] indeed shows a similar structural arrangement at the NTD trimer interfaces (Fig 1C; Cα RMSD values of 2.26 Å and 3.66 Å for VSG615 and VSG1954, respectively). Hence, the *in silico* analysis favors a homotrimeric arrangement for LiTat 1.3 VSG.

### LiTat 1.3 VSG occurs as a homotrimer in solution

Next, we employed various in solution biophysical techniques to validate the *in silico* predictions: analytical gel filtration (AGF), SEC coupled to multi-angle light scattering (SEC-MALS), and small-angle X-ray scattering (SAXS).

**Fig 2:**
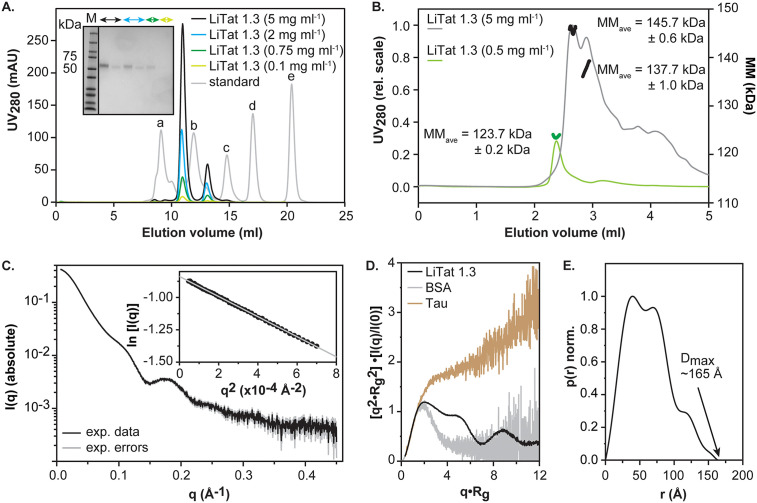
In-solution biophysical characterization of LiTat 1.3. (A.) AGF. The elution profiles of LiTat 1.3 injected at various concentrations onto a Superdex 200 Increase GL 10/300 column are shown in light green (0.1 mg/mL), green (0.75 mg/mL), blue (2 mg/mL), and black (5 mg/mL). SDS-PAGE analyses of the fractions under the collected peaks are shown in the inset. The elution profile of the standard is indicated by the grey line, with elution peaks indicated by small letters: a) bovine thyroglobulin (MM = 670 kDa), b) bovine γ-globulin (MM = 158 kDa), c) chicken ovalbumin (MM = 44 kDa), d) horse myoglobulin (MM = 17 kDa), e) vitamin B12 (MM = 1.35 kDa). (B.) SEC-MALS. The elution profiles (plotted on a relative scale on the first y-axis) of LiTat 1.3 injected at various concentrations onto an Agilent Bio SEC-3 column are shown in green (0.5 mg/mL) and grey (5 mg/mL). The molecular masses determined for the corresponding elution peaks (plotted on an absolute scale on the second Y-axis) are indicated by the green and black dots, respectively. The average molecular masses and their standard deviations are shown for the reader’s convenience. (C.) SAXS. The experimental data and errors are shown as black and grey lines, respectively. The inset displays the Guinier region with the experimental data and the Guinier fit visualized as black dots and a grey line, respectively. (D.) Normalized Kratky plot. The data of LiTat 1.3 VSG, BSA (reference globular protein), and Tau (reference random-coil IDP) are colored black, grey, and brown, respectively. (E.) Pair-distance distribution function. The maximal particle dimension (D_max_) is shown for the reader’s convenience.

AGF was performed with LiTat 1.3 VSG at different concentrations (Fig 2A). Analysis of the chromatograms consistently reveals the existence of two elution peaks with apparent molecular masses (MM_app_) of ~280 kDa and ~100 kDa, respectively. Both elution peaks contain LiTat 1.3 VSG as evidenced by SDS-PAGE analysis (Fig 2A, inset). Interestingly, the first elution peak represents the major elution fraction at all tested concentrations, indicating that LiTat 1.3 VSG most probably adopts a homo-oligomeric architecture in solution. As AGF determines MM_app_ based on hydrodynamic volume, it is inherently sensitive to protein shape and conformational flexibility in solution. Hence, extended and/or highly dynamic conformations lead to MM_app_ estimations that are higher than would be expected based on sequence alone, making it difficult to draw any conclusions on the exact oligomeric state of LiTat 1.3 VSG under the two observed elution peaks. To further investigate the oligomeric state of LiTat 1.3 VSG in solution, we performed SEC-MALS under the same conditions (Fig 2B). SEC-MALS is a technique that enables MM determination on an absolute scale, as it is not influenced by protein shape and/or conformational flexibility. The elution profiles measured at two concentrations show elution peaks with average absolute MM values close to those theoretically expected for a homotrimer, thereby providing strong evidence that LiTat 1.3 VSG occurs as a homotrimer in solution. Interestingly, in both AGF and SEC-MALS experiments, the elution profiles remain quasi unaltered regardless of the tested concentration, suggesting that LiTat 1.3 homotrimer formation is concentration-independent within the tested concentration range.

Finally, the in-solution features of LiTat 1.3 VSG were also probed by SAXS (Fig 2C), which is a (low-resolution) structural technique that is highly suitable for studying the conformational behavior of biological macromolecules in solution [46,47]. A prerequisite for the extraction of structural parameters from a SAXS data set is that the sample under study is ideal (there are no interparticle effects) and mono-disperse (the sample is homogeneous), which can be assessed through Guinier analysis. The experimentally collected LiTat 1.3 VSG scattering curve obtained from the first (major) elution peak is characterized by a long, linear Guinier region, which is strongly indicative of ideal, monodisperse behavior (Fig 2C, inset). A thorough analysis of SAXS-derived structural parameters and MM estimations (S1 Table) are fully consistent with a homotrimer, thereby directly supporting the AGF and SEC-MALS data sets. Interestingly, the SAXS data indicate that the LiTat 1.3 VSG homotrimer is characterized by a significant degree of flexibility. First, normalized Kratky plot analysis (Fig 2D) [48] reveals an in-solution behavior that is intermediate compared to reference curves for random coil intrinsically disordered proteins (IDPs; Tau) and rigid, globular proteins (bovine serum albumin, BSA). While the latter have a bell-shaped appearance with a tail returning to zero, those for random coil IDPs reach a plateau followed by a continuous increase with higher scattering angles. The normalized Kratky plot of LiTat 1.3 displays multiple peaks (consistent with the occurrence of multiple domains and an oligomeric state) and contains a tail that does not return to zero (indicating a certain degree of flexibility). Second, the p(r) plot also suggests a pronounced asymmetry reflected by an elongation ratio larger than 2 (Fig 2E) [49].

In conclusion, these in-solution biophysical studies provide experimental evidence that LiTat 1.3 VSG adopts a homotrimeric, non-globular architecture in solution.

### The solution structure of LiTat1.3 VSG is best described by a conformational ensemble that considers CTD flexibility

**Fig 3:**
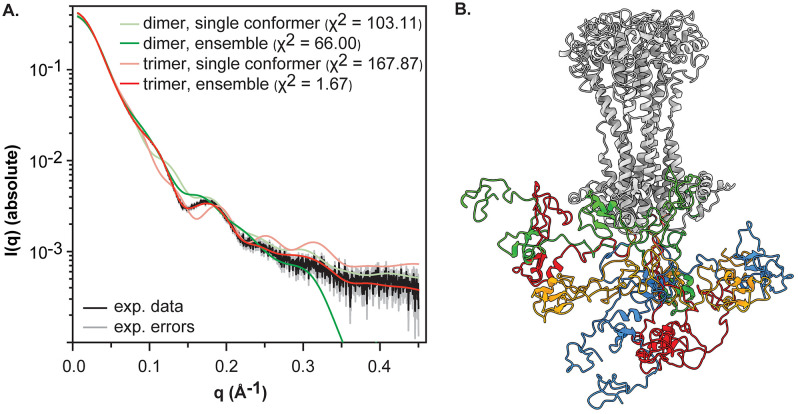
Conformational ensemble of the LiTat 1.3 trimer. (A.) Comparison of the fits for different models to the experimental data. The latter are visualized in black, with the error margins shown in grey as in Fig 2. The fits of the different models are shown in light green (LiTat 1.3 dimer, AlphaFold single conformer), green (LiTat 1.3 dimer, conformational ensemble), salmon (LiTat 1.3 trimer, AlphaFold single conformer), and red (LiTat 1.3 trimer, conformational ensemble). The χ^2^ are reported as a measure for the goodness of fit for the reader’s convenience. (B.) Cartoon representation of the LiTat 1.3 trimer conformational ensemble that best describes the in-solution SAXS data. All conformers have been superposed using the NTD, which is colored grey. The CTD of each conformer is shown in a different color to highlight the CTD’s high flexibility.

Next, the SAXS data were employed to investigate the conformational dynamics of LiTat 1.3 VSG in solution. A first comparison between the experimental scattering curve and a scattering curve calculated for the initial AlphaFold-Multimer single-state model clearly demonstrates that the in-solution behavior of LiTat 1.3 VSG cannot be explained by a single conformer (χ² = 167.87; Fig 3). Together with the above-mentioned biophysical data suggesting that LiTat 1.3 VSG contains a certain degree of flexibility, this indicates that its solution structure is best described by a conformational ensemble. To this end, we relied on SAXS-driven ensemble modeling with BilboMD as previously performed for dimeric VSGs [18]. Here, we assumed that the CTD would be highly flexible / intrinsically disordered given that i) this was suggested by *in silico* prediction (*cfr*. Fig 1) and ii) this behavior has been thoroughly documented for dimeric VSGs [17,18]. The selection of rigid and flexible regions for subsequent ensemble modeling is summarized in S1 Table and based on previously published work [17,18]. Indeed, ensemble modeling that considers a highly flexible CTD provides a significantly better fit (χ² = 1.67), thereby suggesting that LiTat 1.3 adopts a conformational ensemble rather than a single, rigid structure. From the obtained models, it becomes clear that the CTD undergoes substantial structural rearrangements in solution and possesses a conformational freedom akin to the CTDs of dimeric VSGs. For the sake of completion, we calculated scattering curves for both rigid and flexible LiTat 1.3 VSG dimers and compared these to the experimental data set. It again becomes clear that LiTat 1.3 does not occur as a dimer in solution and that the homotrimeric arrangement best explains the data set.

## Discussion

*T. b. gambiense* is the primary causative agent of HAT, a parasitic disease that continues to impose a significant public health burden in sub-Saharan Africa. Accurate diagnosis remains critical for controlling transmission and advancing disease elimination efforts [50]. In this context, the *T. b. gambiense*-specific LiTat 1.3 VSG is a key antigen for serological diagnosis. Indeed, LiTat 1.3 expressing parasites are the sole basis for the Card Agglutination Trypanosomiasis Test (CATT) [51] that is used for massive screening of populations at risk, and it is the major *gambiense*-specific antigen in the individual HAT Sero K-Set rapid diagnostic test (Coris BioConcept) [52]. Despite its critical role in serological gHAT-diagnostics, it remains unclear why LiTat 1.3 specifically is a reliable diagnostic marker for a possible *T. b. gambiense* infection. Resolving the structural and biophysical properties of LiTat 1.3 represents an essential first step towards the understanding of what makes this VSG distinct from others for host immune recognition and serological diagnostic performance. In this study, we comprehensively characterized its structural features, oligomeric state, and conformational flexibility.

A first conclusion from this work is that LiTat 1.3 VSG purified from *in vivo* grown trypanosome BSFs exists as a homotrimer in solution, as evidenced by a combination of biophysical analyses (AGF, SEC-MALS, and SAXS) and structural modeling (SAXS-based conformational modeling using AlphaFold-Multimer models as starting structures). Hence, this work experimentally validates the previous classification of LiTat 1.3 as a class B2 VSG [22]. However, under the tested experimental conditions, LiTat 1.3 appears to maintain its trimeric state, which is in contrast with previously characterized class B2 trimeric VSG-species, where oligomerization was found to be concentration-dependent. It was therefore assumed that, under the extreme packing densities present at the parasite surface, these concentration-dependent VSGs would indeed adopt trimeric assemblies, although direct experimental evidence supporting this assumption has remained lacking [14,53]. In contrast, our data provides the first experimental evidence to date that a VSG like LiTat 1.3 can form stable homotrimers even at low protein concentrations, demonstrating that trimerization in this case reflects an intrinsic property rather than a consequence of molecular crowding. While this does not yet constitute direct evidence of trimer formation on the parasite surface itself, it substantially strengthens the view that B-type VSGs can naturally occur as trimers in the coat. Why LiTat 1.3 retains its trimeric state compared to previously characterized B-type VSG trimers remains unclear. One noteworthy difference between our study and previous reports [14,53] is that a full-length VSG (including the CTD) was analyzed here, in contrast to VSG NTDs obtained through proteolytic removal of the CTD from full-length VSG prior to experimentation. Although NTDs retrieved from proteolytically digested dimeric VSGs are known to form stable dimers in solution, it is not yet known whether VSG trimer formation is fully mediated by the NTD or whether this requires additional stabilizing by the CTDs. Including the CTD in our analysis may have contributed to the stable trimeric state of LiTat 1.3. In any case, working with a full-length VSG can be considered as a more physiologically relevant approach, since this form (including both its NTD and CTD) is naturally present on the parasite surface.

The second main conclusion based on the presented data is that the in-solution behavior of LiTat 1.3 is best described by a conformational ensemble rather than a rigid, singular structure. In this study, we employed an established BilboMD-based workflow to generate such ensembles, while acknowledging that a growing range of complementary ensemble and AlphaFold-informed approaches have recently been developed for modeling flexible or partially disordered proteins (e.g., Ensemblify and AFflecto [54,55]). The resulting LiTat 1.3 ensemble contains both compact and extended conformers that exhibit structural features that mirror those observed in dimeric VSGs; *i.e.*, a rigid, structured NTD and a highly flexible CTD of which its dynamic character is mediated by intrinsic disorder [17,18]. Although CTD conformational flexibility was so far only reported for dimeric VSGs, our findings provide the first experimental evidence that this feature is also present in trimeric VSGs. This demonstrates that CTD dynamics are not limited to dimeric forms and may represent a more general property of the VSG family, regardless of their oligomeric state. Hence, as this observed flexibility is most likely also present on the BSFs surface membrane, it indicates that trimeric VSGs also contribute to the dynamics of the VSG coat [17,18].

These findings suggest that it is highly likely that LiTat 1.3 occurs as a homotrimer on the parasite surface. As GPI valence (number of GPIs per molecule) have been indicated to control VSG-trafficking and turnover in bloodstream *T. brucei*, a trimeric VSG like LiTat 1.3 (with a GPI3 valence) would be expected to be stably associated with the cell surface as the dimeric (GPI2) VSGs [53]. In turn, the homotrimeric state is expected to influence the organization of the VSG coat, its physical and functional properties, and its immunological footprint. First, a coat of VSG trimers could display altered packing densities compared to VSG dimers, thereby modulating “gap sizes” in the VSG coat and thus its shielding of underlying invariant surface proteins from host immune recognition. Second, the trimeric assemblies may influence the overall dynamics and trafficking of VSGs within the coat, which are essential for maintaining a functional, adaptive barrier. In this context, Hartel *et al*. demonstrated that the diffusion coefficient of a GPI-anchored dimeric VSG is inversely related to the axis length of its dimerization interface, with an increase in axis length by one-third resulting in a three-fold reduction of the diffusion coefficient on living trypanosomes [56]. This effect was shown to be an intrinsic property of ectodomain length, and entirely independent of protein-protein interactions or changes in lateral protein density. While the axis length of a homotrimeric VSG is similar to that of a homodimeric VSG, the former’s inherently larger molecular mass and size could also be expected to be inversely related to its diffusion coefficient. In other words, under this hypothesis, a GPI-anchored LiTat 1.3 would exhibit a reduced diffusion coefficient as a consequence of its homotrimeric architecture, which would impact its lateral mobility at the parasite surface [56]. Third, it has been demonstrated that the trypanosome’s forward swimming motion generates hydrodynamic dragging forces on antibody-VSG complexes. These dragging forces drive a backward movement of the latter toward the flagellar pocket, thereby providing a biophysical mechanism for the directional clearance of such complexes [57]. Importantly, it has been demonstrated that clearance rates scale with the size of the antibody-VSG complexes (e.g., IgM-VSG complexes are cleared two to three times faster compared to IgG-VSG complexes) [57]. Hence, compared to a homodimeric architecture, a homotrimeric VSG could therefore be expected to promote the formation of larger (and perhaps even more crosslinked) antibody-VSG complexes, thereby experiencing greater hydrodynamic drag and thus faster directional sorting toward the flagellar pocket [57]. Finally, the occurrence of a VSG trimer may very well serve as a means to expand the concept of antigenic variation with regards to antibody recognition. Indeed, it can be hypothesized that a trimeric architecture generates an antigenic surface that is fundamentally distinct from that of a dimeric VSG implying that a parasite surface coated with VSG trimers displays different immunogenic epitopes exposed to the host immune system compared to a coat composed of VSG dimers. This may explain why LiTat 1.3 elicits a strong antibody response during a chronic *T. b. gambiense* infection rendering it a robust antigen for serological diagnostic testing. However, the assumption that this would solely be ascribed to a difference in oligomeric state is clearly an oversimplification, as this cannot fully account for its diagnostic performance. A perfect counterexample is provided by LiTat 1.5, a dimeric VSG [18] that represents a secondary antigen exploited in gHAT serology, especially in the individual HAT Sero K-Set and Abbott Bioline 2.0 HAT rapid diagnostic tests and the immunotrypanolysis test [52,58,59]. Although the precise biological consequences of a VSG trimer coat remain to be determined, it is interesting that natural infections with *T. b. gambiense* exhibit a strong bias toward the expression of homotrimeric class B VSGs on the parasite surface, a characteristic not observed in other trypanosome species [22]. This species-specific preference possibly hints at a competitive advantage offered to *T. b. gambiense* by trimer VSG in a way that would enable the parasite to sustain its characteristic long-term chronic infections in the human host. Taken together, VSG homotrimers may represent another means of achieving a trade-off between maximal surface coverage, antigenic variation, retaining sufficient mobility to support coat remodeling and turnover, and achieving efficient clearance of antibody-VSG complexes. While these considerations suggest that the trimeric architecture of LiTat 1.3 may have meaningful biological consequences, it should be stressed that these remain speculative in the absence of direct experimental evidence.

In summary, our findings provide new insights into the structural and biophysical properties of LiTat 1.3, the predominant diagnostic VSG for *T. b. gambiense*. These results contribute to the broader understanding of VSG structural diversity, particularly in terms of oligomerization and domain flexibility. By establishing a structural framework for a key diagnostic antigen, this work lays the foundation for future studies exploring the relationships between VSG structure, antigenicity, and diagnostic performance.

## Supporting information

S1 Table SAXS data collection and scattering-derived parameters for LiTat 1.3 VSG. (DOCX)

S1 File Raw Gel. Original uncropped SDS-PAGE gel image corresponding to Fig 2A (inset). Lanes from left to right: molecular weight marker (M), followed by six lanes of purified *T. b. gambiense* LiTat 1.3 VSG at decreasing concentrations (5, 5, 2, 2, 0.75 and 0.1 mg/ml).
(PDF)
