# Supplementary material for: The major diagnostic VSG LiTat 1.3 of the human parasite Trypanosoma brucei gambiense is a trimer in solution
Source: PLoS Pathog. 2026 Aug 10;22(8):e1014530. doi: 10.1371/journal.ppat.1014530 (PMC13480635; doi:10.1371/journal.ppat.1014530)
Supplement: Supplementary file 1 [file ppat.1014530.s002.pdf]

Original uncropped gel image corresponding to Figure 2A (inset)

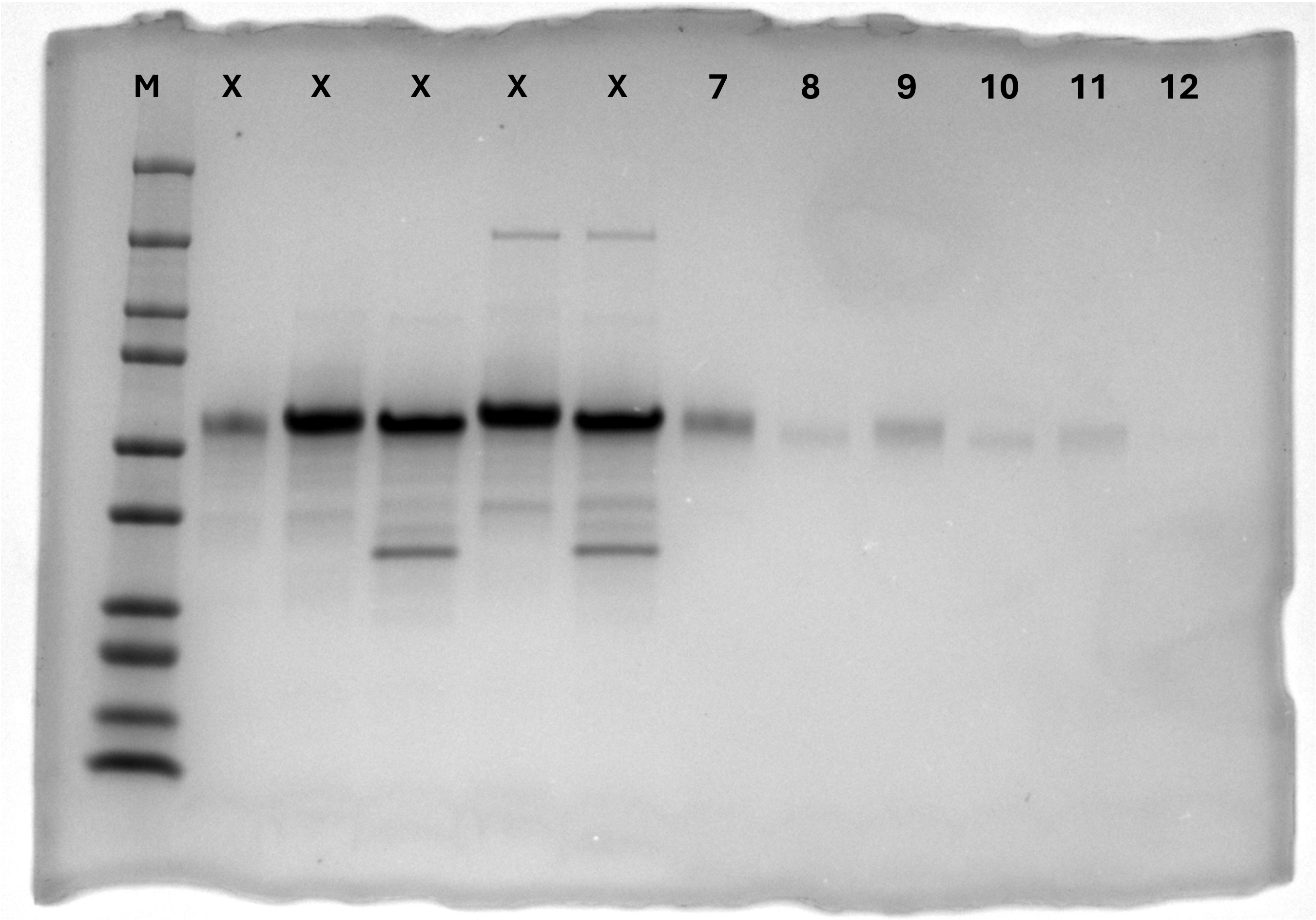

Gel type: SDS-PAGE, 4-15% mini-PROTEAN TGX precast gel (Bio-Rad), run in Tris-Glycine-SDS buffer at 120V. Protein bands were visualized using PageBlue protein staining solution (Thermo Scientific).

Imaging method: Invitrogen iBright CL1500 Imaging System

Loading order and sample identity:

- **Lane M:** Precision Plus Protein Dual Color Standard (Bio-Rad)
- **Lane 2-5:** X (not included in final figure)
- **Lane 7:** *Tbg* LiTat 1.3 (5 mg/ml)
- **Lane 8:** *Tbg* LiTat 1.3 (5 mg/ml)
- **Lane 9:** *Tbg* LiTat 1.3 (2 mg/ml)
- **Lane 10:** *Tbg* LiTat 1.3 (2 mg/ml)
- **Lane 11:** *Tbg* LiTat 1.3 (0.75 mg/ml)
- **Lane 12:** *Tbg* LiTat 1.3 (0.1 mg/ml)

Note: Lanes 2-5 were excluded from the final figure as they were not relevant to the manuscript. These lanes are marked with an X on the raw image.
