# Supplementary material for: The major diagnostic VSG LiTat 1.3 of the human parasite Trypanosoma brucei gambiense is a trimer in solution
Source: PLoS Pathog. 2026 Aug 10;22(8):e1014530. doi: 10.1371/journal.ppat.1014530 (PMC13480635; doi:10.1371/journal.ppat.1014530)
Supplement: Supplementary file 2 [file ppat.1014530.s001.docx]

**Table S1.** SAXS data collection, scattering-derived, and structural modeling parameters for LiTat 1.3 VSG. The table also reports on structural metrics for the final ensemble.

|  | ***T. brucei gambiense* LiTat 1.3 VSG** |
| --- | --- |
| **Data collection parameters** |  |
| Beam line | SWING (SOLEIL) |
| Wavelength (Å) | 0.99 |
| *q* range (Å^-1^)* | 0.00045 – 0.55 |
| Concentration (mg ml^-1^) (mode) | 15.5 (SEC-SAXS) |
| Buffer conditions | 20 mM HEPES, 150 mM NaCl, 3% glycerol, pH 7.4 |
| Temperature (°C) | 20 |
|  |  |
| **Structural parameters^§^** |  |
| *I(0)* (cm^-1^) [from Guinier] | 0.43 |
| R_g_ (Å) [from Guinier] | 48.21 |
| *I(0)* (cm^-1^) [from *p(r)*] | 0.44 |
| R_g_ (Å) [from *p(r)*] | 46.64 |
| E.R. | 2.76 |
| *D_max_* (Å) | ~165 |
| Porod volume estimate, *V_p_* (Å^3^) | 252354 |
| Porod exponent | 3.8 |
|  |  |
| **Molecular mass determination** |  |
| MM (kDa) [from *SAXSMoW* on final merged curve] | 147.00 (*q* = 0.25 Å^-1^) |
| MM (kDa) [from *Q_R_* on final merged curve] | 142.25 (*q* = 0.25 Å^-1^) |
| MM (kDa) [from *V_p_*/1.7] | 148.44 |
| Calculated MM from sequence (kDa) | 144.50 |
|  |  |
| **Ensemble modeling** |  |
| Conformer generation and selection | BILBOMD |
| Protein regions selected to be fixed/rigid | 24-366 (NTD), 384-417 (CTD structured domain 1), 434-457 (CTD structured domain 2) |
|  |  |
| **Software employed** |  |
| Data processing and analysis | ATSAS, BioXTAS RAW |
| Computation of theoretical intensities and fitting | FoXS, MultiFoXS |
| **SASBDB entry** | SASDXA9 |
| **Ensemble metrics** |  |
| End-to-end distance (NTD to CTD; nm) | 7.21 ± 3.22 (conformer 1), 16.27 ± 1.05 (conformer 2), 8.71 ± 0.40 (conformer 3), 9.12 ± 0.63 (conformer 4), 10.33 ± 3.91 (overall) |
| End-to-end distance (core to CTD; nm) | 7.86 ± 1.26 (conformer 1), 8.56 ± 0.64 (conformer 2), 5.59 ± 0.77 (conformer 3), 5.99 ± 0.63 (conformer 4), 7.00 ± 1.51 (overall) |
| End-to-end distance (NTD to core; nm) | 8.48 ± 0.02 (overall) |
| Solvent-accessible surface area (nm^2^) | 743.91 ± 4.87 |
| **PED entry** | accession number pending |

Abbreviations: *I(0)*, extrapolated scattering intensity at zero angle; *R_g_*, radius of gyration calculated using either Guinier approximation (from Guinier) or the indirect Fourier transform package GNOM [from *p(r)*]; *MM*, molecular mass; *D_max_*, maximal particle dimension; *V_p_*, Porod volume; *V_ex_*, particle excluded volume; E.R., elongation ratio

*Momentum transfer |*q*| = 4πsin(θ)/λ
