# Supplementary material for: The major diagnostic VSG LiTat 1.3 of the human parasite Trypanosoma brucei gambiense is a trimer in solution
Source: PLoS Pathog. 2026 Aug 10;22(8):e1014530. doi: 10.1371/journal.ppat.1014530 (PMC13480635; doi:10.1371/journal.ppat.1014530)
Supplement: Supplementary file 3 [file ppat.1014530.s003.docx]

**Reply to the reviewers** **(PNTD-D-25-01929)**

**The major diagnostic VSG LiTat 1.3 of the human parasite *Trypanosoma brucei gambiense* is a trimer in solution**

Niki Danel, Rob Geens, Israel Mares-Mejia, Wim Versées, Jan Van Den Abbeele, Yann G.-J. Sterckx

First and foremost, we would like to thank the reviewers and editorial staff for their thorough review of our manuscript.

**ADDITIONAL EDITOR COMMENTS**

Dear Dr Sterckx,

This paper has been reviewed by three experts in the field of protein biochemistry and biophysics and, one of them also with experience in *Trypanosoma brucei* diagnosis. As you will see from the reviews, the paper was considered technically very sound.

However, two of the reviewers raised concerns about several points that I also consider important to be addressed before the paper can be accepted for publication.

Additionally, the paper deals basically with biochemistry and biophysical information on oligomerization state of the diagnostic VSG LiTat 1.3. Therefore, I believe the paper would better suit the scope of this journal if it could be combined with some kind of immunological analysis.

*Reply:* We thank the Editor for the careful evaluation of our manuscript and for the positive assessment of its technical soundness. We are pleased that, with the reviewers’ constructive remarks, the manuscript has been further strengthened. We have, in our opinion, addressed all concerns raised by the reviewers (none of whom requested additional experimental work).

Despite our best intentions, it is not entirely clear to us which immunological analysis the editor recommends/requests. We have therefore not added new immunological data in the revised manuscript. Importantly, LiTat 1.3 is a key antigen used in *T. brucei gambiense* serodiagnosis, and its oligomeric state and structural organization are directly relevant to antigen presentation and antibody recognition. As highlighted by the reviewers, a detailed understanding of the biochemical and biophysical properties of this diagnostic VSG provides valuable insight for the HAT research and diagnostic community. We believe that, with the clarifications and improvements implemented in response to the reviewers’ comments, the revised manuscript remains fully aligned with the journal’s scope and readership.

**REVIEWER 1**

**Methods**

This manuscript analyses the quaternary structure of LiTat 1.3, which is a trimeric B type VSG. The aim is to see whether it is indeed a trimer, and to get some structural information using SAXS and alpha-fold modeling. This was done on purified, soluble proteins.

**Results**

SEC, SEC-MALS and SAXS methods showed this protein to be a trimer. This is not new and not surprising, as AF3 gives high confident prediction of the trimer, as does Hidden Markov Model (HMM)-based Python pipeline referenced in the paper. In addition, SAXS was used to model the protein, with the best fit obtained when the CTD is flexible, as has also been shown before for the dimeric form. However, previous studies on the dimeric form gave much higher resolutions on the flexibility of the CTD and its function (see for example PMID: 28894098).

*Reply R1.1:* This manuscript represents a follow-up study of the paper “Beyond the VSG layer: exploring the role of intrinsic disorder in the invariant surface glycoproteins of African trypanosomes” by Sülzen et al., previously published in *PLoS Pathogens* (PMID 38648216). During the peer review process of our previous paper, one of the referees raised the intriguing question of how the VSG coat dynamics would be affected in case of a VSG homotrimer. For VSG homodimers, studies on their solution structure have revealed that, while the N-terminal domains (NTDs) behave as rigid entities, the C-terminal domains (CTDs) are hallmarked by a high degree of flexibility, thereby rendering the entire VSG coat highly dynamic. While the same behavior could be expected for homotrimeric VSGs, such evidence was yet to be presented. The fact that the referee raised this question underlines its relevance. In this paper, we directly address this question through the application of an integrative structural biology approach to investigate the solution state of *T. b. gambiense* LiTat 1.3 VSG. The novelty of our study is three-fold.

First, it represents the first experimental structural characterization of a diagnostic *T. b. gambiense* VSG. Second, we demonstrate that LiTat 1.3 forms a stable homotrimer in solution. While both AlphaFold and the HMM-based approaches indeed predict a trimeric organization for LiTat 1.3, these remain computational predictions. Despite the high confidence scores, such models require experimental validation. This is particularly relevant for VSGs, for which the oligomeric state has long been assumed to be dimeric and where computational models alone are insufficient to establish their solution behavior. Through the approach presented here, we experimentally validate the occurrence of concentration-independent, trimeric VSGs. To the best of our knowledge, this is the first report of such an observation. Third, CTD flexibility has indeed been described previously for dimeric VSGs. However, as noted above, it has not been experimentally demonstrated for trimeric VSGs. Our SAXS-based ensemble modeling shows that trimeric LiTat 1.3 exhibits comparable intrinsic CTD flexibility, thereby extending prior observations to a distinct oligomeric class. Hence, trimeric VSGs also contribute to the parasite’s highly dynamic surface antigen coat, which contributes to our understanding of host-trypanosome interactions.

We have revised the Results and Discussion to better emphasize these points and to clearly distinguish computational predictions from experimentally validated structural properties.

**Conclusions**

With current technologies, this paper does not supply sufficient new information to be worth a publication. Many different VSG proteins have been analyzed, including in the trimeric form. See for example ref 14 in this paper. The only reason for this paper to have some interest is that LiTat 1.3 is used for diagnosis of T. b. gambiense infections, and not much structural work has been done on this specific protein. Still, one would expect a more thorough analysis of structure and kinetics of this protein, to be worth publication.

*Reply R1.2:* We respectfully disagree with this assessment. While previous studies have analyzed VSGs with different oligomeric organizations, these were largely limited to other *T. brucei* subspecies. Our study provides the first experimental structural characterization of a *T. b. gambiense* VSG in its full-length form and demonstrates a stable, concentration-independent trimeric assembly in solution. We applied an integrative structural biology approach that represents the current gold standard for simultaneously determining oligomeric state and conformational flexibility of large glycoproteins in solution. Given that LiTat 1.3 is a key antigen used in the serodiagnosis of *T. b. gambiense* infection, we believe that insights into its solution structure and conformational dynamics are relevant for interpreting its diagnostic performance and could guide the improvement/optimization of future diagnostic serological reagents for gHAT.

**Editorial and Data Presentation Modifications**

(No Response)

**Summary and General Comments**

see above

**REVIEWER 2**

**Methods**

The objective of this study is the structural and biophysical characterization of the variant surface glycoprotein (VSG) LiTat1.3 of T. b. gambiense. VSGs and the surface coats they form lie at the interface of parasite and mammalian host and play an essential role in parasite survival and sustained infection. In addition, VSG LITat1.3 is important as it plays a central role in serological screening for gHAT infections which belong to the neglected tropical diseases. The Methods section is structured clearly and follows a clear line of thought.

The authors begin the results part with the classification of the NTD of LiTat1.3 using an available HMM-based Python pipeline. Please acknowledge that this classification of the NTD of LiTat1.3 has already been published in So et al. 2022 (reference 44 in the authors’ manuscript).

*Reply R2.1:* We have revised the results section to explicitly acknowledge that the NTD subtype classification of LiTat 1.3 was previously reported by So et al. 2022, and better frame that our analysis is consistent with this published classification. This sentence now reads as follows:

“*Consistent with earlier work of So et al. 2022 [22], our HMM-based analysis also assigned LiTat 1.3 to the B2 subtype, which harbors trimeric VSGs [14].*”

One difference between the analysis shown here and previous structural analyses such as those shown in Dakovic et al., 2023 is the use of the entire VSG protein instead of the NTD only. For homodimeric VSGs it is known that the NTD on its own forms a stable dimer in solution. I assume the trimerization is determined by the NTD also, but could the presence of the CTDs have an effect on the stability of this trimer in solution?

*Reply R2.2:* We now acknowledge that our use of full-length VSGs differs from previous structural studies based on clipped NTDs and discuss the possibility that the CTD may influence trimer stability in solution. This part now reads as follows:

*“One noteworthy difference between our study and previous reports [14,53] is that a full-length VSG (including the CTD) was analyzed here, in contrast to VSG NTDs obtained through proteolytic removal of the CTD from full-length VSG prior to experimentation. Although NTDs retrieved from proteolytically digested dimeric VSGs are known to form stable dimers in solution, it is not yet known whether VSG trimer formation is fully mediated by the NTD or whether this requires additional stabilizing by the CTDs. Including the CTD in our analysis may have contributed to the stable trimeric state of LiTat 1.3. In any case, working with a full-length VSG can be considered as a more physiologically relevant approach, since this form (including both its NTD and CTD) is naturally present on the parasite surface.”*

Minor suggestions:

The authors use VSG purified from T. b. gambiense by ConA chromatography. It would be helpful to mention whether the obtained VSG is in its soluble or membrane form.

*Reply R2.3:* This concerns soluble VSG and this is now explicitly mentioned in the methods section.

When modelling with AlphaFold the authors state that they removed the N-terminal signal sequence (determined with SignalP6.0). However, the authors did not remove the C-terminal GPI-signal sequence which is cleaved when the GPI-anchor is added to the protein. Would removing this sequence have an impact on the modelling results? Online tools, such as NetGPI1.1 (also available from DTU Health Tech), are available for identifying this signal sequence.

*Reply R2.4:* We have now predicted the C terminal GPI-anchor signal sequence using NetGPI 1.1 and have removed it prior to AlphaFold structure prediction. However, this hasn’t altered the predicted oligomeric arrangement or overall structural conclusions. The method section has been updated accordingly to clearly describe the removal of both the N terminal signal peptide and the C terminal GPI signal sequence prior to modelling. This now reads as follows:

“*Molecular models of LiTat 1.3 dimers and trimers were generated using AlphaFold-Multimer[29] based on the primary amino acid sequence (GenBank accession no. KJ499460.1) [30]. The signal peptide, which is cleaved from the mature VSG protein, was predicted using the SignalP 6.0 tool [31] and excluded from subsequent modeling. In addition, the C-terminal GPI-anchor signal sequence was predicted using NetGPI-1.1 and likewise excluded prior to modeling [32].*”

**Results**

The authors use a combination of methods to convincingly show that VSG LiTat1.3 presents as a homotrimer in solution, and that this is independent of the concentration of the protein. This concentration-independent formation of a trimer has not been shown for a VSG previously. Using SAXS experiments, they also show that trimeric VSGs have an inherent flexibility which is conferred by their CTD, much the same has been reported for dimeric VSGs previously. The results are clearly presented, and the figures are of sufficient quality for clarity.

**Conclusions**

The authors conclude that the LiTat1.3 VSG studied, is a homotrimer in solution and that its conformation is flexible. These are valid conclusions given the results of the experiments shown. They discuss these findings mainly in the context of the role of LiTat1.3 in gHAT diagnosis and touch upon VSG coat dynamics.

The discussion appears to lack clarity and a clear train of thought, though. The presence of homotrimeric (stable in solution, independent of protein concentration in the case of LiTat1.3) and not solely homodimeric VSGs must has huge ramifications on the nature of the surface coat. I wonder for instance what effect homotrimers will have on the packing/density and the mobility of the coat.

*Reply R2.5:* We have substantially reorganized the discussion. In the revised version, we first retain a short general introduction to frame the biological and diagnostic context. We then explicitly present the two principal conclusions of our work. First, we establish that LiTat 1.3 forms a concentration-independent homotrimer and briefly discuss possible contributions of the CTD to this stability. Second, we demonstrate that the CTD is conformationally flexible in solution. Building on these findings, we subsequently explore what these properties may imply for the parasite surface. We now discuss how trimeric organization may influence coat architecture, packing, shielding of invariant proteins, mobility, and antibody engagement.

We believe that this reorganization provides a clearer narrative flow and makes the broader significance of our findings more apparent.

**Editorial and Data Presentation Modifications**

At present the manuscript is difficult to read as the motivation for this study does not really become apparent until the end of the manuscript. Some inconsistencies regarding the state of knowledge on the oligomerization state of VSGs throughout the manuscript also add to some confusion at Initial reading of the manuscript. I therefore suggest that the authors should review and adjust the manuscript to make the motivation and relevance of this work clearer from the start and to eliminate seemingly inaccurate and contradictory statements.

The abstract, in particular, would benefit from a few more words on the background so that the question the authors are pursuing is clear from the start. Also, the authors state in the abstract that they “present evidence that LiTat1.3 ….. displays a homotrimeric architecture in solution instead of the archetypal homodimeric structure expected for a VSG.” Whereas the finding of a concentration-independent VSG trimer is novel, it has been shown previously that the ‘archetypal homodimers’ are only formed by A-type, not B-type VSGs (Dakovic et al., 2023). LiTat1.3 was already classified as a B2-type VSG in So et al., 2022, prior to the work presented here. Only later in the manuscript do the authors acknowledge that B-type VSGs have already been shown not to form the archetypal homodimers.

*Reply R2.6:* We thank the reviewer for highlighting these points. We have revised both the abstract and the introduction to make the motivation and relevance of our study clearer from the outset. Specifically, we now explicitly state that, while LiTat 1.3 was previously classified as a B2-type VSG and B-type VSGs have been suggested to form trimers, no experimental evidence for LiTat 1.3’s oligomeric state existed prior to this work. We also clarified that the concentration-independent formation of a homotrimer, as observed here, is a novel feature compared to previously studied B-type VSGs. These changes remove the previous ambiguities regarding the state of knowledge on VSG oligomerization and more clearly frame the question and significance of our study.

Minor suggestions

In the Author’s Summary the authors state that HAT is caused by Trypanosoma brucei gambiense. Though this is the major causative agent, this is not the only subspecies of T. brucei that causes HAT. The authors may want to rephrase to reflect this.

l. 72: “VSG are further categorised ….” The categorisation into A- and B-type is based on the NTDs alone, not the entire VSG, this should therefore read: VSG NTDs are further categorised ...

l. 79: The authors state that VSG NTDs “adopt a myriad of distinct topologies”.

Does the structural classification of T. brucei VSGs not rather suggest that there are a finite number of distinct topologies which are the basis of the classification (A1-A3 and B1-B2) of the VSGs?

l. 82: “iii) VSGs may adopt distinct oligomeric states (thereby challenging the longstanding paradigm that VSGs solely occur as homodimers)”

To my knowledge a previous study (Dakovic et al., 2023) has shown convincingly that B-type VSGs do not form the stable homodimers found for A-type VSGs.

ll. 95-97: “While LiTat 1.5 was recently characterized as an archetypical dimeric class A1 VSG [18], the structural details of LiTat 1.3 remain poorly understood.”

LiTat 1.3 has been previously classified as having a B2-type NTD (So et al., 2022). The authors should acknowledge this (s. Methods).

l. 184: Wyatt Technology

l. 339 chronic infections

*Reply R2.7:* The manuscript has been carefully revised to incorporate all the suggested minor corrections and clarifications.

**Summary and General Comments**

Trypanosoma brucei gambiense is the major of the two causative agents of the neglected tropical disease known as human African trypanosomiasis or sleeping sickness which is lethal if untreated. An important feature of African trypanosomes is their dense surface coat that consists of variant surface glycoprotein. This coat acts as a protective layer and undergoes antigenic variation to allow the parasite to evade the adaptive immune system and maintain an infection. One of these VSGs, LiTat1.3, is used as a major diagnostic marker for serological screening to identify human infections with T. b. gambiense. The properties of this protein are not known. The authors therefore embark on a characterisation of this VSG by a combination of structural modelling, biophysical techniques and low-resolution structural analysis and they convincingly show that LiTat1.3 – independent of concentration, which is novel – forms a stable homotrimer in solution. Using SAXS experiments they also show that this trimeric VSG displays the same inherent flexibility as has been shown previously for homodimeric VSGs.

As mentioned above, the motivation for analysing the structural characteristics of LiTat1.3 and the significance of the results could be presented more clearly in the manuscript.

The fact that LiTat1.3 is a homotrimer in solution, even at low concentration, is the so far clearest validation of the notion that B-type VSGs form homotrimers. This poses interesting questions as to the formation and functioning of this protective surface coat which is important for establishing and maintaining an infection. The properties of the coat (packing, diffusion, antigenicity, …) should likely differ depending on whether a dimeric or trimeric VSG is being expressed.

On the other hand, LiTat1.3 is an important diagnostic marker. The manuscript would benefit from a more focused and detailed discussion of the potential impact of the findings presented in the manuscript. What concrete impact do the authors believe the improved structural understanding might have on improving future diagnostic tools.

*Reply R2.8:* we refer to *Reply R2.5* as an answer to this question.

**REVIEWER 3**

**Methods**

The central, testable hypothesis is that VSG LiTat 1.3 forms a stable homotrimer in solution, directly challenging the established VSG dimeric paradigm.

The integrative approach (AlphaFold, SEC-MALS, and SEC-SAXS with ensemble modeling) is the gold standard for robustly determining the oligomerization state and flexibility of large solution-state proteins.

The subject, VSG LiTat 1.3, is a crucial diagnostic antigen, making its structural characterization highly relevant

The high power is confirmed by the strong internal consistency: three independent SAXS-derived molecular mass estimates (147.00, 142.25, 148.44 kDa) align closely with the calculated trimer mass (144.50 kDa). However, the computational power (800 conformers) needs explicit confirmation of sampling sufficiency (a minor revision point).

*Reply R3.1:* There appears to have been a misunderstanding with regard to the BilboMD pipeline. BilboMD does not generate a structural pool of only 800 conformers for ensemble generation and selection but computes 800 conformers per Rg bin. With a total of 6 Rg bins, this comes down to a structural pool of 4800 conformers. Hence, BilboMD offers a sufficiently high computational power.

We have nonetheless verified whether a larger structural pool would affect the modeling outcome by combining the conformers from independent BilboMD Classic runs into a single superpool and performing collective analysis using BilboMD Multi. This approach did improve the fit to the experimental SAXS data, as indicated by a lower χ². We clarified it in the manuscript as follows:

*“BilboMD Classic was used to generate conformational ensembles by sampling six R_g_ bins, with minimal and maximal R_g_ values set at 7% and 35% of the experimentally determined R_g_, respectively. For each R_g_ bin, 800 conformations were generated, yielding 4800 conformers per BilboMD Classic run. In total, three independent BilboMD Classic runs were performed, and the resulting pools were subsequently combined into a single super-pool (14400 conformers) from which a minimal ensemble was selected using BilboMD Multi and the experimental scattering curve [35].”*

Standard biophysical analyses, including Guinier and p(r) plots, are used, and the low final chi^2 ~2 for ensemble fitting rigorously supports the conclusions.

Ethical/Regulatory concerns: I believe is Not Applicable. The study is an in vitro structural characterization of a recombinant protein.

*Reply R3.2:* The ethics statement is applicable as the LiTat 1.3 VSG used in this study was obtained from parasites cultivated in rats (and not from a recombinant source). Accordingly, the study involved ethical approval for the use of animals to culture *T. b. gambiense* and purify the VSG.

**Results**

The analyses conducted follow the plan described in the Methods, progressing logically from AlphaFold-Multimer predictions to SEC-MALS validation and SAXS-based structural characterization.

The results are clearly and coherently presented, with appropriate reporting of key parameters such as Rg, Dmax, molecular mass estimates, and χ² values. The transition from the poor fit of the rigid AlphaFold model to the excellent agreement obtained through ensemble modeling is especially well explained and strongly supports the conclusions regarding CTD flexibility. However. The details regarding the modelling results are not fully described.

Figures are of high quality, with clear scattering curves, Guinier and Kratky plots, and well-rendered structural models.

*Reply R3.3:* We have added more details regarding the modelling in the methods section, which now reads as follows:

*“SAXS-based ensemble modelling was carried out using BilboMD [34,35]. All simulations were performed using the default BilboMD pipeline. Briefly, conformational sampling was performed using the standard CHARMM-based implementation. Following energy minimization, high-temperature molecular dynamics simulations at 1500 K were performed in vacuum, with only the atoms of the flexible domains allowed to move. The resulting conformers were validated using FoXS followed by minimal ensemble selection with MultiFoXS [36–38]*.”

**Conclusions**

The conclusions are well supported by the data presented. The combination of SEC-MALS, multiple SAXS-derived molecular mass estimates, and ensemble modeling provides strong and internally consistent evidence that LiTat 1.3 forms a stable homotrimer in solution. The substantial improvement in SAXS fit when moving from a rigid AlphaFold model to an ensemble-based description convincingly demonstrates flexibility within the C-terminal domains. The interpretations are careful, biologically plausible, and aligned with the experimental observations.

The limitations of the analysis are partly described. While the authors note the general challenges associated with modeling flexible domains, the manuscript would benefit from a clearer explanation of the assumptions underlying the ensemble modeling approach, including the definition of rigid and flexible segments and the inherent dependence on sampling strategies. These clarifications would strengthen transparency but do not undermine the main conclusions.

The authors effectively discuss how their findings advance our understanding of VSG structure and diversity. Demonstrating that a major diagnostic VSG adopts a trimeric architecture expands the known repertoire of VSG oligomerization states and provides new insight into structural variation within the surface coat of T. brucei. The work also highlights the role of CTD flexibility, extending observations previously limited to dimeric VSGs.

Public health relevance is addressed appropriately. Because LiTat 1.3 is a key antigen used in serological diagnosis of T. b. gambiense infection, a structural explanation of its organization and dynamics is directly relevant for understanding diagnostic performance and may inform future reagent development.

*Reply R3.4:* we refer to *Reply R3.3* as an answer to this question.

**Editorial and Data Presentation Modifications**

To further strengthen the clarity, rigor, and reproducibility of the ensemble modeling, the following technical points regarding the BilboMD/MultiFoXS methodology must be clarified and detailed:

1- Rationale for Flexible/Rigid Domains: Readers would benefit from a clearer explanation of how the AlphaFold-Multimer metrics—specifically pLDDT and Predicted Aligned Error (PAE)—guided your identification of the fixed/rigid segments (residues 24-366, 384-417, and 434-457) and the flexible linkers. This rationale is key to the BilboMD setup and should be explicitly presented. Although BilboMD is an entirely appropriate choice for your ensemble generation, you may consider a brief mention of related flexible-modeling or AlphaFold-based ensemble tools—such as Ensemblify, IDPConformerGenerator, or AFflecto—to situate your approach within the growing ecosystem of computational methods for modeling flexible or partially disordered proteins.

*Reply R3.5:* The selection of rigid and flexible regions for ensemble modeling is based on previously published work of Bartossek (2017) and Sülzen (2023) and is now specifically mentioned. We also described other ensemble tools in the discussion: *“In this study, we employed an established BilboMD-based workflow to generate such ensembles, while acknowledging that a growing range of complementary ensemble and AlphaFold-informed approaches have recently been developed for modeling flexible or partially disordered proteins (e.g. Ensemblify and AFflecto [54,55]).”*

2- Simulation Parameters: A concise description of the essential BilboMD/CHARMM simulation settings (e.g., force field, solvent model, simulation temperature, and approximate sampling length) would make the modeling strategy fully transparent without requiring extensive methodological expansion.

*Reply R3.6:* All simulations were performed using the default BilboMD pipeline. As mentioned earlier, we have now added more details regarding the modelling in the methods section.

3- Ensemble Selection Mechanism: Because ensemble modeling approaches differ substantially, please explicitly state whether the final SAXS-compatible ensemble resulted from a subset-selection approach (MultiFoXS minimal ensemble search, selecting a few representative structures) or a reweighting approach (such as Bayesian/Maximum Entropy reweighting, assigning weights to a large number of conformers). This clarification will help readers interpret the structural heterogeneity implied by the model.

*Reply R3.7:* All simulations were performed using the default BilboMD pipeline, where FoXS and MultiFoXS are used. We further refer to *Reply R3.3* as an answer to this question.

4 - Sampling Sufficiency: A brief comment indicating that the pool of 800 conformers adequately sampled the relevant conformational landscape—for example, by noting the range of Rg and Dmax values or the structural diversity present—would further reinforce the thoroughness of your modeling. For disordered regions 800 poses could be a small number. The pool size is very often in the thousands range. With alternative approaches (such as Ensemblify, IDPConformerGenerator, or AFflecto), you might increase the pool size.

*Reply R3.8:* we refer to *Reply R3.3* as an answer to this question.

5- Intrinsic Disorder Validation (Strong Recommendation): While not required, a complementary intrinsic disorder prediction using a tool like IUPred2A, PrDOS, or Metapredict should be performed and presented. Showing that sequence-based predictors reach the same conclusion regarding the high flexibility of the CTD regions would provide an important, independent line of evidence supporting the ensemble model.

*Reply R3.9:* We thank the reviewer for this suggestion and fully agree. We have performed complementary sequence-based intrinsic disorder predictions. All predictors identify the CTD of LiTat 1.3 as intrinsically disordered. These results are now included in Figure 1 and are mentioned in the results section as an independent line of evidence supporting the SAXS-based ensemble model. This part reads as follows:

*“Interestingly, the CTDs are characterized by significantly lower pLDDT scores, which implies that these may be intrinsically disordered [45]. This was further assessed by performing sequence-based intrinsic disorder predictions using multiple algorithms (****Fig. 1B****). All predictors identify the CTD of LiTat 1.3 as highly disordered, whereas the NTD is predominantly predicted to be structured. “*

Additional suggestions:

6- Expand the discussion of trimer stability: Your data indicate unusually stable, concentration-independent trimerization—a brief comparative analysis with previous B2 VSG interfaces.

*Reply R3.10:* We have expanded the discussion to better position the concentration independent stability of LiTat 1.3 relative to previously described B2 VSGs. At present, the precise reason for the enhanced stability of this trimer remains unknown. One possible explanation is that, in contrast to earlier studies that focused on proteolytically derived N terminal domains, our analysis includes the full-length protein. It therefore remains unresolved whether trimer formation is fully mediated by the NTD or whether additional contacts involving the CTDs contribute to stabilization. We note that inclusion of the CTD also provides a more physiologically relevant view, since both domains are present in the native parasite coat. The manuscript now reads as follows:

“*Why LiTat 1.3 retains its trimeric state compared to previously characterized B-type VSG trimers remains unclear. One noteworthy difference between our study and previous reports [14,53] is that a full-length VSG (including the CTD) was analyzed here, in contrast to VSG NTDs obtained through proteolytic removal of the CTD from full-length VSG prior to experimentation. Although NTDs retrieved from proteolytically digested dimeric VSGs are known to form stable dimers in solution, it is not yet known whether VSG trimer formation is fully mediated by the NTD or whether this requires additional stabilizing by the CTDs. Including the CTD in our analysis may have contributed to the stable trimeric state of LiTat 1.3. In any case, working with a full-length VSG can be considered as a more physiologically relevant approach, since this form (including both its NTD and CTD) is naturally present on the parasite surface.*”

7- Discuss the potential influence of purification conditions: A sentence noting that ConA-based extraction maintains native VSG quaternary structure (as reported in prior work) will reassure readers that trimerization is not an artifact.

*Reply R3.11:* We have added a sentence in the methods section noting that ConA-based purification is widely used for soluble VSG and does not alter its native quaternary structure, supporting that the observed trimer reflects the protein’s native state. This sentence reads as follows:

“*The next day, the VSG-containing supernatant was loaded onto a 1 mL HiTRAP ConA 4B column (Cytiva) at a flow rate of 0.5 mL/min, after which the column was washed with 10 column volumes (CVs) of ConA binding buffer, and soluble VSGs was eluted using ConA elution buffer (ConA binding buffer supplemented with 10% methyl α-D-mannopyranoside). ConA affinity chromatography is a routinely used strategy for isolating soluble native VSG from trypanosome surface coats and does not change the native quaternary structure of the protein [23].*”

8- The role of CTD intrinsic disorder could be further discussed, as well as its structural landscape.

*Reply R3.12:* The discussion includes the following part:

“*The resulting LiTat 1.3 ensemble contains both compact and extended conformers that exhibit structural features that mirror those observed in dimeric VSGs; i.e., a rigid, structured NTD and a highly flexible CTD of which its dynamic character is mediated by intrinsic disorder [17,18]. Although CTD conformational flexibility was so far only reported for dimeric VSGs, our findings provide the first experimental evidence that this feature is also present in trimeric VSGs. This demonstrates that CTD dynamics are not limited to dimeric forms and may represent a more general property of the VSG family, regardless of their oligomeric state. Hence, as this observed flexibility is most likely also present on the BSFs surface membrane, it indicates that trimeric VSGs also contribute to the dynamics of the VSG coat [17,18].*”

**Summary and General Comments**

This manuscript provides a structural and biophysical characterization of the Variant Surface Glycoprotein (VSG) LiTat 1.3, an antigen of diagnostic importance in Trypanosoma brucei gambiense infections. The combination of AlphaFold-Multimer predictions, SEC-MALS, and SEC-SAXS data—interpreted through ensemble modeling—results in a coherent demonstration that LiTat 1.3 forms a stable homotrimer in solution.

From previous structural work on VSGs, the dominant paradigm has been the dimer, and the evidence presented here clearly establishes that LiTat 1.3 represents a distinct trimeric class. The SAXS-based ensemble modeling provides compelling evidence for substantial CTD flexibility, extending previous observations made for dimeric VSGs.

Conclusion:

This is a well-executed and scientifically valuable study. The discovery that LiTat 1.3 forms a stable trimer in solution is a contribution to our understanding of VSG diversity and parasitic surface architecture. With minor clarifications to the ensemble modeling methodology, the manuscript will be stronger and more accessible to readers.

*Reply R3.13:* We refer to all replies formulated above.
