# Supplementary material for: The major diagnostic VSG LiTat 1.3 of the human parasite Trypanosoma brucei gambiense is a trimer in solution
Source: PLoS Pathog. 2026 Aug 10;22(8):e1014530. doi: 10.1371/journal.ppat.1014530 (PMC13480635; doi:10.1371/journal.ppat.1014530)
Supplement: Supplementary file 4 [file ppat.1014530.s004.docx]

**Reply to the reviewers** **(PPATHOGENS-D-26-01144)**

**The major diagnostic VSG LiTat 1.3 of the human parasite *Trypanosoma brucei gambiense* is a trimer in solution**

Niki Danel, Rob Geens, Israel Mares-Mejia, Wim Versées, Jan Van Den Abbeele, Yann G.-J. Sterckx

First and foremost, we would like to thank the reviewers and editorial staff for their thorough review of our manuscript.

**Journal Requirements**

If the reviewer comments include a recommendation to cite specific previously published works, please review and evaluate these publications to determine whether they are relevant and should be cited. There is no requirement to cite these works unless the editor has indicated otherwise.

1) Please amend your detailed Financial Disclosure statement. This is published with the article. It must therefore be completed in full sentences and contain the exact wording you wish to be published.

i) State the initials, alongside each funding source, of each author to receive each grant. For example: "This work was supported by the National Institutes of Health (####### to AM; ###### to CJ) and the National Science Foundation (###### to AM).". If you did not receive any funding for this study, please simply state: u201cThe authors received no specific funding for this work.u201d

*Reply:* We have amended the detailed Financial Disclosure statement accordingly.

2) Thank you for stating "The SAXS data have been deposited to the SASBDB (<https://www.sasbdb.org/>) with the accession code SASDXA9." However, we were unable to access the sequence data using the information provided. Please review and update the Data Availability Statement to ensure that the underlying data are publicly accessible and that all accession numbers, links, or repository details are correct and functional.

*Reply:* All data related to this entry will be released upon publication of the manuscript. This is similar to the release of a structure in the PDB and is considered standard practice in the field. At the reviewer’s suggestions, we have also deposited our data in the Protein Ensemble Database (*cfr. Reply R3.1*).

**REVIEWER 2**

**Methods**

(No Response)

**Results**

(No Response)

**Conclusions**

(No Response)

**Editorial and Data Presentation Modifications**

(No Response)

**Summary and General Comments**

The authors have carefully addressed the reviewers‘ comments, which has improved the quality of the manuscript. Most of my concerns have been resolved, with only a few points remaining:

1) In the Author’s abstract the statement “In our study, we discovered that, unlike most VSGs that form pairs of identical molecules (homodimers), LiTat 1.3 assembles into groups of three (homotrimers). “still seems too strong, as it has been known for several years that not all VSGs form homodimers. This should be toned down appropriately.

*Reply R2.1:* We agree that the original wording was still too strong in the Author’s abstract. The statement has been revised to avoid implying that LiTat 1.3 is the first non-dimeric VSG described.

2) While the structural findings presented in this manuscript are interesting, the implications for coat biology and advantages for diagnostics are still underdeveloped. The authors may wish to strengthen this point.

*Reply R2.2:* We expanded the Discussion section further. Specifically, we now relate the homotrimeric architecture of LiTat 1.3 to previous studies on VSG lateral mobility and antibody clearance, discussing how the larger size of its ectodomain (compared to dimeric VSGs) could potentially influence diffusion at the parasite surface. In addition, we discuss how the presentation of three epitopes per trimer may promote the formation of larger antibody-antigen complexes, which could affect hydrodynamic sorting toward the flagellar pocket. However, we explicitly state that these considerations are highly speculative and that these hypotheses are based on existing literature. We conclude that dedicated experimental studies will be required to establish the impact of the trimeric oligomeric state on VSG coat biology. This section now reads as follows:

*Second, the trimeric assemblies may influence the overall dynamics and trafficking of VSGs within the coat, which are essential for maintaining a functional, adaptive barrier. In this context, Hartel et al. demonstrated that the diffusion coefficient of a GPI-anchored dimeric VSG is inversely related to the axis length of its dimerization interface, with an increase in axis length by one-third resulting in a three-fold reduction of the diffusion coefficient on living trypanosomes [56]. This effect was shown to be an intrinsic property of ectodomain length, and entirely independent of protein-protein interactions or changes in lateral protein density. While the axis length of a homotrimeric VSG is similar to that of a homodimeric VSG, the former’s inherently larger molecular mass and size could also be expected to be inversely related to its diffusion coefficient. In other words, under this hypothesis, a GPI-anchored LiTat 1.3 would exhibit a reduced diffusion coefficient as a consequence of its homotrimeric architecture, which would impact its lateral mobility at the parasite surface [56]. Third, it has been demonstrated that the trypanosome’s forward swimming motion generates hydrodynamic dragging forces on antibody-VSG complexes. These dragging forces drive a backward movement of the latter toward the flagellar pocket, thereby providing a biophysical mechanism for the directional clearance of such complexes [57]. Importantly, it has been demonstrated that clearance rates scale with the size of the antibody-VSG complexes (e.g., IgM-VSG complexes are cleared two to three times faster compared to IgG-VSG complexes)[57]. Hence, compared to a homodimeric architecture, a homotrimeric VSG could therefore be expected to promote the formation of larger (and perhaps even more crosslinked) antibody-VSG complexes, thereby experiencing greater hydrodynamic drag and thus faster directional sorting toward the flagellar pocket [57].*

3) In adding references to the manuscript, references 18 and 56 appear to refer to the same publication (bioRxiv preprint and peer-reviewed version, respectively). These can likely be merged to one citation, citing the peer-reviewed publication only.

*Reply R2.3*: We thank the reviewer for identifying this duplication. The references have been merged, and only the peer reviewed version is now cited throughout the manuscript.

**REVIEWER 3**

**Methods**

The Methods are well suited to the path leading to the results and are clearly explained. No further comments.

**Results**

Minor/suggestions comments:

#1 The SAXS data was deposited in a dedicated repository, the SASBDB (https://www.sasbdb.org), which is the standard database for SAXS data. For the generated structural ensembles, we suggest a similar approach, using either Protein Ensemble Database (https://proteinensemble.org) or Zenodo, to ensure accessibility and long-term availability.

*Reply R3.1:* Following the reviewer's recommendation, we have now also made the generated structural ensembles publicly available through the PED. The corresponding accession information has been added to the manuscript in Table S1. We also explicitly mention this in the Materials and Methods section under “Small-angle X-ray scattering and ensemble modeling”, of which the final sentences now read as follows:

*The information on data collection, scattering-derived structural parameters, and modeling is summarized in Supplementary Table 1. All SAXS data have been deposited in the SASBDB [37] under accession code SASDXA9. The structural ensemble has been submitted to the Protein Ensemble Database (PED) [38], and the accession number is currently pending. It will be added to the manuscript upon assignment. Specific ensemble metrics are also reported in Supplementary Table 1.*

#2 The ensemble metric descriptors could be included to properly to characterize generated ensembles. Ensemble properties/Global features (beyond the radius of gyration), such as end‐to‐end distance, and solvent‐accessible surface area; local features include dihedral angles, flexibility indices, and residue‐level order parameters etc. distance C-Terminus to the core trimer could be a plus.

*Reply R3.2:* We have now included additional ensemble descriptors to provide a more comprehensive characterization of the generated structural ensembles. These metrics have been incorporated into the Results section and Table S1 (also see *Reply R3.1*). All other metrics and experimental details are publicly available through the SASBDB and PED accession numbers.

**Conclusions**

The conclusions are well presented and constitute an important contribution to the field.

**Editorial and Data Presentation Modifications**

Accept (with Minor Revisions)**Summary and General Comments**

This manuscript presents a rigorous and well-executed structural analysis of the Variant Surface Glycoprotein LiTat 1.3, a key diagnostic antigen for Trypanosoma brucei gambiense. By combining AlphaFold-Multimer predictions with SEC-MALS and high-quality SAXS data analyzed through ensemble modeling, the authors convincingly demonstrate that LiTat 1.3 adopts a stable homotrimeric architecture in solution and exhibits substantial flexibility in its C-terminal domains. These findings challenge the prevailing dimer-centric view of VSG organization and expand our understanding of VSG structural diversity. The study is clearly written, methodologically sound, and supported by high-quality data and figures.

All my minor clarifications regarding ensemble modeling methodology were convincingly answered.

I added some minor suggestions, but they are not a prerequisite for recommending this paper for publication.

**REVIEWER 4**

**Methods**

The manuscript of Danela et al ("The major diagnostic VSG LiTat 1.3 of the human parasite Trypanosoma brucei gambiense is a trimer in solution") presents a biochemical, structural (SAXS), and modeling analysis of VSG LiTat 1.3, a protein central to rapid diagnosis of T. b. gambiense, as this VSG is an early coat protein in infection and the host develops a detectable antibody response to it. The data presented makes a very strong case for the main hypothesis of the paper: namely, that VSG LiTat 1.3 is a homotrimer in solution.

**Results**

yes x 3

**Conclusions**

I have come "late" to the review of this manuscript and have seen the other reviewers' critiques and the author responses. I believe that the authors have more than adequately addressed the technical and scientific points raised, and that, with their data and the preponderance of other biochemical and structural data on the B-class VSGs (of which VSG LiTat 1.3 is a clear member), the conclusion of the paper is quite solid.

**Editorial and Data Presentation Modifications**

(No Response)

**Summary and General Comments**

The manuscript of Danela et al ("The major diagnostic VSG LiTat 1.3 of the human parasite Trypanosoma brucei gambiense is a trimer in solution") presents a biochemical, structural (SAXS), and modeling analysis of VSG LiTat 1.3, a protein central to rapid diagnosis of T. b. gambiense, as this VSG is an early coat protein in infection and the host develops a detectable antibody response to it. The data presented makes a very strong case for the main hypothesis of the paper: namely, that VSG LiTat 1.3 is a homotrimer in solution.

I have come "late" to the review of this manuscript and have seen the other reviewers' critiques and the author responses. I believe that the authors have more than adequately addressed the technical and scientific points raised, and that, with their data and the preponderance of other biochemical and structural data on the B-class VSGs (of which VSG LiTat 1.3 is a clear member), the conclusion of the paper is quite solid.
