# Supplementary material for: The major diagnostic VSG LiTat 1.3 of the human parasite Trypanosoma brucei gambiense is a trimer in solution
Source: PLoS Pathog. 2026 Aug 10;22(8):e1014530. doi: 10.1371/journal.ppat.1014530 (PMC13480635; doi:10.1371/journal.ppat.1014530)
Supplement: Peer review history

# Author comment: Author response to Decision Letter 0

**DOI:** 10.1371/journal.ppat.1014530.r001

**Transfer Alert**

This paper was transferred from another journal. As a result, its full editorial history (including decision letters, peer reviews and author responses) may not be present.

5 Jun 2026

Attachment Submitted filename: Response_to_reviewers_comments_FINAL.docx

# Editor report: Decision Letter 0

**DOI:** 10.1371/journal.ppat.1014530.r002

**Authors:** Dominique Soldati-Favre

12 Jun 2026

PPATHOGENS-D-26-01144

The major diagnostic VSG LiTat 1.3 of the human parasite Trypanosoma brucei gambiense is a trimer in solution

PLOS Pathogens

Dear Dr. Sterckx,

Thank you for submitting your manuscript to PLOS Pathogens. After careful consideration, we feel that it has merit but does not fully meet PLOS Pathogens's publication criteria as it currently stands. Therefore, we invite you to submit a revised version of the manuscript that addresses the points raised during the review process.

Please address the reviewers' comments that were shared with you in the editor decision letter of May 7th PNTD-D-25-01929R1 and pasted below,

Please submit your revised manuscript by Jul 12 2026 11:59PM. If you will need more time than this to complete your revisions, please reply to this message or contact the journal office at plospathogens@plos.org. When you're ready to submit your revision, log on to https://www.editorialmanager.com/ppathogens/ and select the 'Submissions Needing Revision' folder to locate your manuscript file.

Please include the following items when submitting your revised manuscript:

* A letter that responds to each point raised by the editor and reviewer(s). You should upload this letter as a separate file labeled 'Response to Reviewers'. This file does not need to include responses to any formatting updates and technical items listed in the 'Journal Requirements' section below.

* A marked-up copy of your manuscript that highlights changes made to the original version. You should upload this as a separate file labeled 'Revised Manuscript with Track Changes'.

* An unmarked version of your revised paper without tracked changes. You should upload this as a separate file labeled 'Manuscript'.

If you would like to make changes to your financial disclosure, competing interests statement, or data availability statement, please make these updates within the submission form at the time of resubmission. Guidelines for resubmitting your figure files are available below the reviewer comments at the end of this letter.

As the corresponding author, your ORCID iD is verified in the submission system and will appear in the published article. PLOS supports the use of ORCID, and we encourage all coauthors to register for an ORCID iD and use it as well. Please encourage your coauthors to verify their ORCID iD within the submission system before final acceptance, as unverified ORCID iDs will not appear in the published article. Only the individual author can complete the verification step; PLOS staff cannot verify ORCID iDs on behalf of authors.

We look forward to receiving your revised manuscript.

Kind regards,

Dominique Soldati-Favre

Section Editor

PLOS Pathogens

Sumita Bhaduri-McIntosh

Editor-in-Chief

PLOS Pathogens

orcid.org/0000-0003-2946-9497

Michael Malim

Editor-in-Chief

PLOS Pathogens

orcid.org/0000-0002-7699-2064

**Additional Editor Comments (if provided):**

**Journal Requirements:**

If the reviewer comments include a recommendation to cite specific previously published works, please review and evaluate these publications to determine whether they are relevant and should be cited. There is no requirement to cite these works unless the editor has indicated otherwise.

1) Please amend your detailed Financial Disclosure statement. This is published with the article. It must therefore be completed in full sentences and contain the exact wording you wish to be published.

i) State the initials, alongside each funding source, of each author to receive each grant. For example: "This work was supported by the National Institutes of Health (####### to AM; ###### to CJ) and the National Science Foundation (###### to AM).".

If you did not receive any funding for this study, please simply state: u201cThe authors received no specific funding for this work.u201d

2) Thank you for stating "The SAXS data have been deposited to the SASBDB (https://www.sasbdb.org/) with the accession code SASDXA9." However, we were unable to access the sequence data using the information provided. Please review and update the Data Availability Statement to ensure that the underlying data are publicly accessible and that all accession numbers, links, or repository details are correct and functional.

**Reviewers' Comments:**

**Methods**

-Are the objectives of the study clearly articulated with a clear testable hypothesis stated?

-Is the study design appropriate to address the stated objectives?

-Is the population clearly described and appropriate for the hypothesis being tested?

-Is the sample size sufficient to ensure adequate power to address the hypothesis being tested?

-Were correct statistical analysis used to support conclusions?

-Are there concerns about ethical or regulatory requirements being met?

Reviewer #2: (No Response)

Reviewer #3: The Methods are well suited to the path leading to the results and are clearly explained. No further comments.

Reviewer #4: The manuscript of Danela et al ("The major diagnostic VSG LiTat 1.3 of the human parasite Trypanosoma brucei gambiense is a trimer in solution") presents a biochemical, structural (SAXS), and modeling analysis of VSG LiTat 1.3, a protein central to rapid diagnosis of T. b. gambiense, as this VSG is an early coat protein in infection and the host develops a detectable antibody response to it. The data presented makes a very strong case for the main hypothesis of the paper: namely, that VSG LiTat 1.3 is a homotrimer in solution.

**********

**Results**

-Does the analysis presented match the analysis plan?

-Are the results clearly and completely presented?

-Are the figures (Tables, Images) of sufficient quality for clarity?

Reviewer #2: (No Response)

Reviewer #3: Minor/suggestions comments:

#1 The SAXS data was deposited in a dedicated repository, the SASBDB (https://www.sasbdb.org), which is the standard database for SAXS data. For the generated structural ensembles, we suggest a similar approach, using either Protein Ensemble Database (https://proteinensemble.org) or Zenodo, to ensure accessibility and long-term availability.

#2 The ensemble metric descriptors could be included to properly to characterize generated ensembles. Ensemble properties/Global features (beyond the radius of gyration), such as end‐to‐end distance, and solvent‐accessible surface area; local features include dihedral angles, flexibility indices, and residue‐level order parameters etc. distance C-Terminus to the core trimer could be a plus.

Reviewer #4: yes x 3

**********

**Conclusions**

-Are the conclusions supported by the data presented?

-Are the limitations of analysis clearly described?

-Do the authors discuss how these data can be helpful to advance our understanding of the topic under study?

-Is public health relevance addressed?

Reviewer #2: (No Response)

Reviewer #3: The conclusions are well presented and constitute an important contribution to the field.

Reviewer #4: I have come "late" to the review of this manuscript and have seen the other reviewers' critiques and the author responses. I believe that the authors have more than adequately addressed the technical and scientific points raised, and that, with their data and the preponderance of other biochemical and structural data on the B-class VSGs (of which VSG LiTat 1.3 is a clear member), the conclusion of the paper is quite solid.

**********

**Editorial and Data Presentation Modifications?**

Use this section for editorial suggestions as well as relatively minor modifications of existing data that would enhance clarity. If the only modifications needed are minor and/or editorial, you may wish to recommend “Minor Revision” or “Accept”.

Reviewer #2: (No Response)

Reviewer #3: Accept (with Minor Revisions)

Reviewer #4: (No Response)

**********

**Summary and General Comments**

Use this section to provide overall comments, discuss strengths/weaknesses of the study, novelty, significance, general execution and scholarship. You may also include additional comments for the author, including concerns about dual publication, research ethics, or publication ethics. If requesting major revision, please articulate the new experiments that are needed.

Reviewer #2: The authors have carefully addressed the reviewers‘ comments, which has improved the quality of the manuscript. Most of my concerns have been resolved, with only a few points remaining:

1) In the Author’s abstract the statement “In our study, we discovered that, unlike most VSGs that form pairs of identical molecules (homodimers), LiTat 1.3 assembles into groups of three (homotrimers). “ still seems too strong, as it has been known for several years that not all VSGs form homodimers. This should be toned down appropriately.

2) While the structural findings presented in this manuscript are interesting, the implications for coat biology and advantages for diagnostics are still underdeveloped. The authors may wish to strengthen this point.

3) In adding references to the manuscript, references 18 and 56 appear to refer to the same publication (bioRxiv preprint and peer-reviewed version, respectively). These can likely be merged to one citation, citing the peer-reviewed publication only.

Reviewer #3: This manuscript presents a rigorous and well-executed structural analysis of the Variant Surface Glycoprotein LiTat 1.3, a key diagnostic antigen for Trypanosoma brucei gambiense. By combining AlphaFold-Multimer predictions with SEC-MALS and high-quality SAXS data analyzed through ensemble modeling, the authors convincingly demonstrate that LiTat 1.3 adopts a stable homotrimeric architecture in solution and exhibits substantial flexibility in its C-terminal domains. These findings challenge the prevailing dimer-centric view of VSG organization and expand our understanding of VSG structural diversity. The study is clearly written, methodologically sound, and supported by high-quality data and figures.

All my minor clarifications regarding ensemble modeling methodology were convincingly answered.

I added some minor suggestions, but they are not a prerequisite for recommending this paper for publication.

Reviewer #4: The manuscript of Danela et al ("The major diagnostic VSG LiTat 1.3 of the human parasite Trypanosoma brucei gambiense is a trimer in solution") presents a biochemical, structural (SAXS), and modeling analysis of VSG LiTat 1.3, a protein central to rapid diagnosis of T. b. gambiense, as this VSG is an early coat protein in infection and the host develops a detectable antibody response to it. The data presented makes a very strong case for the main hypothesis of the paper: namely, that VSG LiTat 1.3 is a homotrimer in solution.

I have come "late" to the review of this manuscript and have seen the other reviewers' critiques and the author responses. I believe that the authors have more than adequately addressed the technical and scientific points raised, and that, with their data and the preponderance of other biochemical and structural data on the B-class VSGs (of which VSG LiTat 1.3 is a clear member), the conclusion of the paper is quite solid.

[NOTE: If reviewer comments were submitted as an attachment file, they will be attached to this email and accessible via the submission site. Please log into your account, locate the manuscript record, and check for the action link "View Attachments". If this link does not appear, there are no attachment files.]

**Figure resubmission:**

**Reproducibility:**

To enhance the reproducibility of your results, we recommend that authors of applicable studies deposit laboratory protocols in protocols.io, where a protocol can be assigned its own identifier (DOI) such that it can be cited independently in the future. Additionally, PLOS ONE offers an option to publish peer-reviewed clinical study protocols. Read more information on sharing protocols at https://plos.org/protocols?utm_medium=editorial-email&utm_source=authorletters&utm_campaign=protocols

# Author comment: Author response to Decision Letter 1

**DOI:** 10.1371/journal.ppat.1014530.r003

22 Jul 2026

Attachment Submitted filename: Response_to_reviewers_v10072026.docx

# Editor report: Decision Letter 1

**DOI:** 10.1371/journal.ppat.1014530.r004

**Authors:** Dominique Soldati-Favre

26 Jul 2026

Dear Prof. dr. ir. Sterckx,

We are pleased to inform you that your manuscript 'The major diagnostic VSG LiTat 1.3 of the human parasite Trypanosoma brucei gambiense is a trimer in solution' has been provisionally accepted for publication in PLOS Pathogens.

Before your manuscript can be formally accepted you will need to complete some formatting changes, which you will receive in a follow up email. A member of our team will be in touch with a set of requests.

Please note that your manuscript will not be scheduled for publication until you have made the required changes, so a swift response is appreciated.

IMPORTANT: The editorial review process is now complete. PLOS will only permit corrections to spelling, formatting or significant scientific errors from this point onwards. Requests for major changes, or any which affect the scientific understanding of your work, will cause delays to the publication date of your manuscript.

Should you, your institution's press office or the journal office choose to press release your paper, you will automatically be opted out of early publication. We ask that you notify us now if you or your institution is planning to press release the article. All press must be co-ordinated with PLOS.

Thank you again for supporting Open Access publishing; we are looking forward to publishing your work in PLOS Pathogens.

Best regards,

Dominique Soldati-Favre

Section Editor

PLOS Pathogens

Dominique Soldati-Favre

Section Editor

PLOS Pathogens

Sumita Bhaduri-McIntosh

Editor-in-Chief

PLOS Pathogens

orcid.org/0000-0003-2946-9497

Michael Malim

Editor-in-Chief

PLOS Pathogens

orcid.org/0000-0002-7699-2064

***********************************************************

Reviewer Comments (if any, and for reference):

# Editor report: Acceptance letter

**DOI:** 10.1371/journal.ppat.1014530.r005

**Authors:** Dominique Soldati-Favre

Dear Prof. dr. ir. Sterckx,

We are delighted to inform you that your manuscript, "The major diagnostic VSG LiTat 1.3 of the human parasite Trypanosoma brucei gambiense is a trimer in solution," has been formally accepted for publication in PLOS Pathogens.

We have now passed your article onto the PLOS Production Department who will complete the rest of the pre-publication process. All authors will receive a confirmation email upon publication.

The corresponding author will soon be receiving a typeset proof for review, to ensure errors have not been introduced during production. Please review the PDF proof of your manuscript carefully, as this is the last chance to correct any scientific or type-setting errors. Please note that major changes, or those which affect the scientific understanding of the work, will likely cause delays to the publication date of your manuscript. Note: Proofs for Front Matter articles (Pearls, Reviews, Opinions, etc...) are generated on a different schedule and may not be made available as quickly.

Soon after your final files are uploaded, the early version of your manuscript, if you opted to have an early version of your article, will be published online. The date of the early version will be your article's publication date. The final article will be published to the same URL, and all versions of the paper will be accessible to readers.

For Research Articles, you will receive an invoice from PLOS for your publication fee after your manuscript has reached the completed accept phase. If you receive an email requesting payment before acceptance or for any other service, this may be a phishing scheme. Learn how to identify phishing emails and protect your accounts at https://explore.plos.org/phishing.

Thank you again for supporting open-access publishing; we are looking forward to publishing your work in PLOS Pathogens.

Best regards,

Sumita Bhaduri-McIntosh

Editor-in-Chief

PLOS Pathogens

orcid.org/0000-0003-2946-9497

Michael Malim

Editor-in-Chief

PLOS Pathogens

orcid.org/0000-0002-7699-2064
